# Functional co-activation of the default mode network in APOE *ε*4-carriers: A replication study ^✩,✩✩^

**DOI:** 10.1016/j.neuroimage.2021.118304

**Published:** 2021-06-27

**Authors:** Lara J. Mentink, João P.O.F.T. Guimarães, Myrthe Faber, Emma Sprooten, Marcel G.M. Olde Rikkert, Koen V. Haak, Christian F. Beckmann

**Affiliations:** a Department of Geriatrics, Radboudumc Alzheimer Centre, Radboud University Medical Center, Nijmegen, The Netherlands; b Donders Institute for Brain, Cognition and Behaviour, Radboud University Medical Center, Nijmegen, The Netherlands; c Department of Communication and Cognition, Tilburg Center for Cognition and Communication, Tilburg University, Tilburg, The Netherlands; d Department of Human Genetics, Radboud University Medical Center, Nijmegen, The Netherlands; e Centre for Functional MRI of the Brain (FMRIB), Nuffield Department of Clinical Neurosciences, Wellcome Centre for Integrative Neuroimaging, University of Oxford, Oxford, United Kingdom

**Keywords:** Alzheimer’s disease, APOE, Polygenic risk score, Default mode network, Replication study

## Abstract

Structural and functional alterations of the brain in persons genetically at-risk for Alzheimer’s disease (AD) are crucial in unravelling AD development. Filippini et al. found that the default mode network (DMN) is already affected in young APOE *ε*4-carriers, with increased co-activation of the DMN during rest and increased hippocampal task activation. We aimed to replicate the early findings of Filippini et al, using the APOE gene, still the principal AD risk gene, and extended this with a polygenic risk score (PRS) analysis for AD, using the Human Connectome Project dataset (HCP). We included participants from the HCP S1200 dataset (age range: 22-36 years). We studied morphometric features, functional DMN co-activation and functional task activation of recollection performance. Permutation Analysis of Linear Models (PALM) was used to test for group differences between APOE *ε*4-carriers and non-carriers, and to test the association with PRS. PALM controls for biases induced by the family structure of the HCP sample. Results were family-wise error rate corrected at *p* < 0.05. Our primary analysis did not replicate the early findings of Filippini et al. (2009). However, compared with non-carriers, APOE *ε*4-carriers showed increased functional activation during the encoding of subsequently recollected items in areas related to facial recognition (p<0.05, t>756.11). This increased functional activation was also positively associated with PRS (APOE variants included) (p<0.05, t>647.55). Our results are supportive for none to limited genetic effects on brain structure and function in young adults. Taking the methodological considerations of replication studies into account, the true effect of APOE *ε*4-carriership is likely smaller than indicated in the Filippini paper. However, it still holds that we may not yet be able to detect already present measurable effects decades before a clinical expression of AD. Since the mechanistic pathway of AD is likely to encompass many different factors, further research should be focused on the interactions of genetic risk, biomarkers, aging and lifestyle factors over the life course. Sensitive functional neuroimaging as used here may help disentangling these complex interactions.

## 1 Introduction

Dementia is a syndrome that encompasses a set of symptoms, such as cognitive decline and behavioural changes, reducing a person’s ability to perform activities of daily living (Winblad et al., 2016). In the ageing population, the number of older adults with dementia is expected to rise (Patterson, 2018). Worldwide, 50 million people are currently living with dementia and this number is predicted to triple by 2050, making dementia a global health priority (Patterson, 2018; Wortmann, 2012). Alzheimer’s disease (AD) is the main cause of dementia, accounting for 50-70% of dementia cases (Hendrie, 1998; Winblad et al., 2016).

High heritability of late-onset AD was reported by twin studies (estimated between 60% and 78%) and by SNP-based approaches (estimated between 12% and 53%), with the apolipoprotein E (APOE) gene, thus forming an important genetic risk factor (Anttila et al., 2018; Farrer et al., 1997; Gatz et al., 1997, 2006; Lee et al., 2012; Liu et al., 2013; Ridge et al., 2016, 2013). The APOE gene has three common alleles, *ε*2, *ε*3 and *ε*4, with frequencies in the general population of 6%, 78% and 15%, respectively (Eisenberg et al., 2010). The *ε*4-allele is genetically associated with late-onset AD, with an odds ratio (OR) of to 3.2 for *ε*4 heterozygotes and an OR of 12.5 for *ε*4 homozygotes, compared to *ε*3 homozygotes (Corder et al., 1993; Farrer et al., 1997; Liu et al., 2013). The *ε*2-allele is thought to be a protective factor for developing AD (OR 0.6 compared to *ε*3 homozygotes) (Corder et al., 1993; Farrer et al., 1997; Liu et al., 2013). In addition to the APOE gene, multiple other genetic variants have been identified in genome-wide association studies that significantly modify the risk of AD (Desikan et al., 2015; Guerreiro et al., 2013; Jansen et al., 2019; Jonsson et al., 2012; Lambert et al., 2013; Liu et al., 2017; Sims et al., 2017).

Previous neuroimaging studies have reported structural and functional alterations in middle-aged and older APOE *ε*4-carriers, such as decreased hippocampal volume, decreased entorhinal cortex thickness and altered resting-state network activation (Bookheimer and Burggren, 2009; Filippini et al., 2009; Trachtenberg et al., 2012; Yu et al., 2014). Resting state networks (RSN) consist of spatially distinct brain regions that show synchronous temporal signal fluctuations during rest, which can be measured with fMRI (Beckmann et al., 2005; Raichle et al., 2001). A specific RSN is the default mode network (DMN), which comprises the anterior cingulate cortex, posterior cingulate cortex, precuneus, medial prefrontal cortex, retrosplenial cortex, parietal cortex and mediotemporal lobe regions (Hafkemeijer et al., 2012). The DMN is known to be affected in neurodegenerative processes; regions of the DMN are among the first to show amyloid-beta deposition and vulnerability to atrophy (Buckner et al., 2005; Greicius et al., 2004; Jones et al., 2011; Palmqvist et al., 2017). Deactivation of the DMN increases during transition from normal aging to mild cognitive impairment (MCI) and AD (Hafkemeijer et al., 2012). Possible alterations of the DMN in young adults genetically at-risk for AD are crucial in unravelling AD development, before confounding factors of older age and AD itself are present.

Filippini et al. reported that the DMN is already affected in young APOE *ε*4-carriers (Filippini et al., 2009). Specifically, using resting-state fMRI, the authors found an increase in DMN co-activation in the medial temporal lobe regions and medial prefrontal and retrosplenial cortices. In addition, fMRI activation during a novel vs. familiar memory-retrieval paradigm was increased in the hippocampal region for APOE *ε*4-carriers compared to non-carriers. Filippini et al. suggested that these results could reflect a compensation mechanism. No differences were found in total gray matter volume and hippocampal volume (Filippini et al., 2009). Even though this study only assessed a small sample size of 18 APOE *ε*4-carriers and 18 matched controls, it has become a landmark paper with many neuroscience papers citing this finding to illustrate the trajectory of functional alterations of the DMN in APOE *ε*4-carriers from young adults and normal aging to MCI and AD (Damoiseaux et al., 2012; Hafkemeijer et al., 2012; Sperling et al., 2010). While multiple studies have been performed on DMN activation in older adults and adults with MCI or AD, there are few studies on the effect of APOE *ε*4 on the DMN in young, healthy adults, of which Filippini et al. is the most highly cited (Filippini et al., 2009; Hafkemeijer et al., 2012; Hodgetts et al., 2019; Su et al., 2015).

In this paper, we aimed to replicate the results found by Filippini et al. using the dataset of the Human Connectome Project (HCP) and additionally combine a broad variety of genetic variants that confer risk for AD in a polygenic risk score (PRS) to study the mechanism between genetic risk and AD development in a more sensitive manner (; ). Our study benefited from a large sample size, advanced data quality and additional improved preprocessing methods, especially regarding data denoising. Filippini et al. established state-of-the-art statistical procedures for resting state connectivity analyses, which are still used today. We adhered to these methods as much as possible. Results of the memory-encoding paradigm could not be replicated directly, but the HCP dataset contains a working memory task combined with a post-hoc item recognition task that was used as an indirect replication; using a different memory task to replicate previous findings adds more confidence to the generalisability of the findings than a direct replication.

The HCP dataset represents the population at large and therefore enabled us to not only replicate the Filippini paper with substantially increased power, but also to study the polygenic nature of any effects and the generalisability of these results, as a broad spectrum of behavioural and demographic variations can be observed in the HCP cohort.

For our study we hypothesise the following:

1. No association of gray matter volumes in young adults with APOE *ε*4 genotypes or with PRS.
2. Increased co-activation of the DMN during rest, in the medial temporal lobe regions and medial prefrontal and retrosplenial cortices specifically, in APOE *ε*4-carriers and participants with a high PRS.
3. Increased hippocampal activation during encoding, with a 0-back task, leading to recollection at the item recognition task, in APOE *ε*4-carriers and participants with a high PRS.

## 2 Methods

### 2.1 Replication

Advances in MRI data acquisition and analyses necessitate deviations from the exact methods used by Filippini et al. The HCP acquired high quality MR data, which should have had little impact on replication results. The standard minimal preprocessing of the HCP data differed on several points: The standard minimal preprocessing included spatial distortion correction, global intensity normalisation, smoothing using 2mm full-width half-maximum (FWHM) instead of 6mm FWHM and artefact removal using FSL’s ICA-based artefact removal (ICA+FIX). The improved preprocessing resulted in less noise in the data, which were advantageous for replication results. The data was stored in CIFTI files, instead of the more common NIFTI file type. CIFTI files store the data as grayordinates instead of voxels, therefore the data could be handled as surface data instead of volume data, which also improved group-based results with reduced noise and more precise spatial correspondence between subjects (see (Glasser et al., 2013)).

Furthermore, the memory performance was indirectly assessed as the same task was not available in the HCP database. In the study of Filippini et al., participants encoded eight images before the task fMRI. During the task fMRI, both familiar and novel items were shown. Main contrast of interest was the task activation of novel vs. familiar images. In our study, the encoding of the images was performed during the task fMRI, and the memory task was performed afterwards. We used the memory task results to identify items that were successfully or unsuccessfully encoded, as well as successfully encoded items that were recollected versus just familiar, and compared the encoding activity between these item-groups. Even though the task activation was measured in a different part of the memory paradigm, we deemed this analysis as relevant, as hippocampal activation is present in periods of both encoding and memory recall (Bookheimer et al., 2000). In addition to this, Filippini et al. investigated the resting brain perfusion using perfusion MRI. However, perfusion MRI is not available in the HCP dataset and perfusion results could therefore not be replicated. Any other deviations from the methods used in Filippini et al. were indicated as such.

### 2.2 Participants

**Fig. 1:**
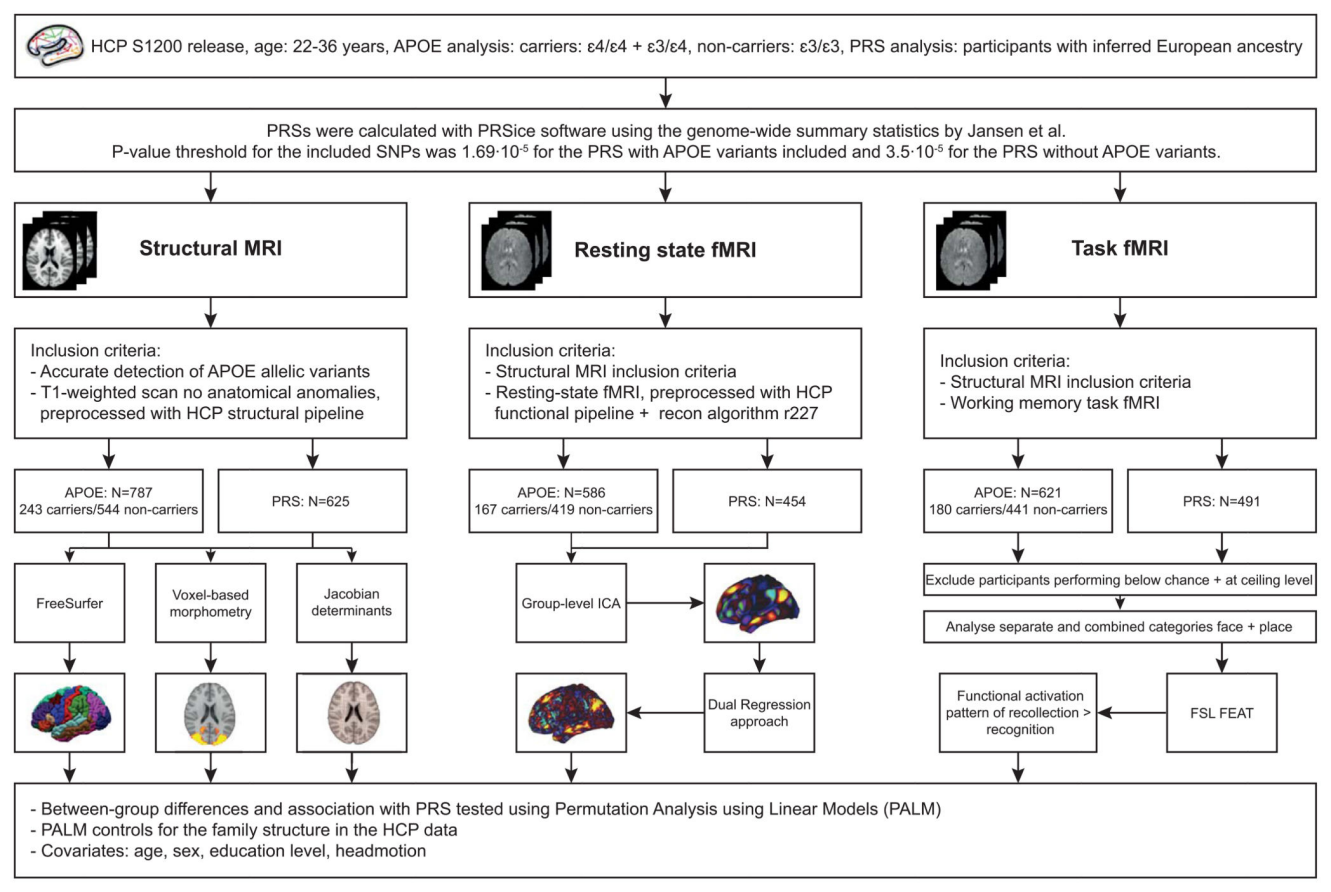
Schematic overview of the analyses.

We used the dataset collected as part of the WU-Minn HCP (S-1200 release), consisting of close to 1200 healthy young adults in the age range of 22-35 years (Van Essen et al., 2013). The HCP aimed for a sample representing the population at large, which allowed us to study whether the effect found by Filippini et al. generalises to a large population. We included participants if the following data were available: complete demographic information, a T1-weighted structural scan, four fully completed 3T resting-state fMRI scans and a completed working-memory task fMRI scan. We excluded participants with anatomical anomalies and segmentation and surface errors in the data (Quality issues A+B) and if the MR images were not reconstructed using the improved image reconstruction algorithm (r227). Participants with at least one *ε*4-allele were included as carriers, participants with two *ε*3-alleles as non-carriers. Participants with the *ε*2-allele were not included, because of the protective effects of the *ε*2-allele (Corder et al., 1994). These in- and exclusion criteria led to a sample size ranging from 454 to 787 participants for the different analyses (Fig. 1).

### 2.3 Neuroimaging protocol

Participants were scanned on a 3T MR scanner with a standard 32-channel head coil at Washington University (Connectome Skyra, Siemens, Germany). The neuroimaging protocol consisted of structural MRI, resting-state fMRI and task fMRI (Human Connectome Project, 2017; Van Essen et al., 2013).

#### 2.3.1 Structural MRI

A T1-weighted MRI scan was acquired using a magnetization-prepared rapid gradient echo sequence (TR = 2400 ms, TR = 2.14 ms, flip angle = 8, field of view = 224 mm, voxel size = 0.7 mm isotropic, acquisition time = 7 min 40 sec) (Human Connectome Project, 2017).

#### 2.3.2 Resting-state fMRI

Resting-state fMRI data were acquired in four runs. The resting-state images were acquired using a gradient-echo EPI sequence (TR =720 ms, TE = 33.1 ms, flip angle = 52, field of view = 208 × 180 mm, voxel size = 2.0 mm isotropic, multiband factor = 8, acquisition time=14 min 33 sec per run) (Human Connectome Project, 2017; Smith et al., 2013).

#### 2.3.3 Task fMRI

The task images for working memory were acquired in two runs (LR and RL phase encoding direction) using a gradient-echo EPI sequence, with 405 frames per run (TR =720 ms, TE = 33.1 ms, flip angle = 52, field of view = 208 × 180 mm, voxel size = 2.0 mm isotropic, multiband factor = 8, acquisition time = 5 min 1 sec per run) (Human Connectome Project, 2017).

#### 2.3.4 Encoding task

The participants performed a working memory task (N-back task with 2-back and 0-back levels) during the task fMRI scan with four stimulus types (faces, places, tools and body parts), shown in different blocks (for detailed information see Barch et al., 2013). After the scan session the participants performed a “Remember, Know, New” item recognition test outside of the scanner. Participants were tested on the faces and places presented during the task in the scanner, with an equal number (48) of new faces and places. Body parts and tools were not included as there were not enough new items. Participants were asked for each item if they had seen it before (old vs. new) and, in case of an old item, whether they could recollect the context of the item (remember) or not (know). Recollection (remembering vs. know) is thought to rely on hippocampus-dependent processes, while recognition (old vs. new) is associated with areas such as the perirhinal cortex (Davachi et al., 2003; Eichenbaum et al., 2007; Eldridge et al., 2000). In our analysis, we will focus on the 0-back condition to minimise any confound of performing a working memory task during the encoding phase.

### 2.4 Data pre-processing

The HCP data has already been preprocessed by the WU-Minn HCP consortium, to ensure data quality. Preprocessing steps are described in short, detailed information about the different pipelines can be found in Glasser et al., 2013 and Smith et al., 2013.

#### 2.4.1 Structural MRI

The structural MR pipeline consisted of three parts; the Pre-FreeSurfer, FreeSurfer and PostFreeSurfer pipelines. In short, the Pre-FreeSurfer pipeline included: undistorted native structural volume space production, T1w and T2w alignment, bias field correction and MNI registration. In the FreeSurfer pipeline, the images were downsampled from 0.7 mm isotropic to 1 mm isotropic and FreeSurfer’s recon-all was run. The PostFreeSurfer pipeline included producing NIFTI (volume) and GIFTI (surface) files, applying surface registration and downsampling the registered surfaces. The recon-all analysis in FreeSurfer used automated labeling for segmentation of the T1w image into 37 neuroanatomical structures and calculated gray matter volumes (mm^3^). Endpoints of interest were total brain volume, gray matter volume, white matter volume and MTL structures (hippocampus, parahippocampal cortex, perirhinal cortex and entorhinal cortex (Fischl, 2012; Fischl et al., 2002). As the FreeSurfer pipeline determined gray matter volumes, we also determined the gray matter density with FSL’s voxel-based morphometry (FSL-VBM). With the Jacobian determinants calculated to register the structural scan to MNI152, we determined local shape differences.

#### 2.4.2 Resting-state and task fMRI

Preprocessing steps of the functional MR data included: spatial distortions correction, head motion correction with FMRIB’s Linear Image Registration Tool (FLIRT), registration to T1-weighted structural image, resampling to MNI space using 2mm FWHM surface smoothing, global intensity normalization, minimal high-pass filtering and artefact removal using ICA + FIX. For additional head motion correction, we determined the frame-wise displacement for each participant using the realignment parameters and excluded participants with a mean root-mean-square of the frame-wise displacement > 0.5 (Oldehinkel et al., 2019). Further, we checked for between-group differences. The cortical surface timeseries and subcortical volume time series were combined in a grayordinate dense time series file (CIFTI, see (Glasser et al., 2013)).

#### 2.4.3 DMN identification

For the resting-state fMRI 25 RSNs on group-level were determined with an independent component analysis approach, using group ICA, implemented in FSL’s MELODIC (Multivariate Exploratory Linear Optimised Decomposition into Independent Components) (Jenkinson et al., 2012). The subject-specific spatial maps were estimated with the dual regression technique (Nickerson et al., 2017); First, the group-level RSNs were used as regressors against each subject’s fMRI dataset in a multivariate regression analysis to estimate the individual timecourses corresponding to each group-level RSN. Second, these individual timecourses were used as regressors against each subject’s fMRI dataset in a second multivariate regression analysis to estimate the subject-specific spatial maps (Beckmann et al., 2009). The preprocessed files contain 25 subject-specific spatial maps for each participant. The spatial map representing the DMN contains the anterior cingulate cortex, posterior cingulate cortex, precuneus, medial prefrontal cortex, retrosplenial cortex, parietal cortex and mediotemporal lobe regions and was selected based on visual inspection of the group ICA results. The DMNs of all participants were combined in one 4D file and separated in left and right hemisphere data using Connectome Workbench (Marcus et al., 2013).

#### 2.4.4 Genetic analyses

Genotyping data was extracted from blood samples using the Illumina Multi-Ethnic Global Array (MEGA) SNP-array, including chip-specific content from PsychChip and ImmunoChip. The data was made available by the HCP via the dbGAP repository (https://www.ncbi.nlm.nih.gov/projects/gap/cgi-bin/study.cgi? study_id=phs001364.v1.p1). We used the genotyping data to determine the APOE genotype in the sample. Further, we ran a PRS analysis, as described by Purcell et al. (Purcell et al., 2009). Quality control of genotyping was carried out by excluding SNPs based on minor allele frequency (MAF < 5%), genotyping call rate (GCR < 95%) and Hardy-Weinberg equilibrium (HWR < 10e-6). We selected for further PRS analysis individuals determined to be from European descent using multi-dimensional scaling analysis with the European 1000 Genomes dataset as reference^1^; the first four components from multi-dimensional scaling analysis on individuals of European descent were tested on association with the MRI outcome variables using PALM (5000 permutations) and added as a covariate under the condition that they reported a significant association (Price et al., 2006; Purcell et al., 2007). Further, genotypes were imputed with the European 1000 Genomes reference panel using RICOPILI, which included additional quality control procedure with thresholding for the quality of the imputation (INFO < 0.1), minor allele frequency (MAF < 5%), genotyping call rate (GCR < 80%), and missing rate (MR < 0.01) (Lam et al., 2019). The PRS was calculated for each participant given the effect sizes from previously published discovery data, using the PRSice software (Jansen et al., 2019; Euesden et al., 2014). The PRS was computed as the sum of the risk-alleles carried by each individual in the HCP sample, weighted by their effect size, whereby both risk-allele and effect size were derived from the original (discovery) GWAS. The formula for the PRS, given OR-based effects and *n* SNPs is:

$$\sum_{SNP=1}^{n}\log\left(OR_{SNP}\right)*X_{SNP}$$

where *X* is the number of risk alleles for the individual in the target sample (here the HCP sample).

We defined the imputed genotyping data reported by HCP individuals of inferred European descent as our target sample, and the genome-wide summary statistics of AD in European subjects reported by Jansen et al. as the discovery sample, which is one of the largest GWAS for AD to date (Jansen et al., 2019). We used standard clumping in PRSice to adjust for linkage disequilibrium. We ran two PRS analyses: one with and one without the APOE allelic variants and their (correlated) variants in linkage disequilibrium (chr19: 45020859–45844508). The P-value threshold for the included SNPs was based on the findings by Jansen et al. (2019); 1.69∙10^−5^ for the PRS with APOE variants included and 3.5∙10^−5^ for the PRS without APOE variants^2^. With the two PRS results, we were able to determine how much of the genetic variance in the imaging phenotypes is explained with and without APOE.

### 2.5 Statistical analyses

#### 2.5.1 Structural MRI

APOE *ε*4-carriers and non-carriers were tested for differences in brain volumes, gray matter density and local shape differences (Jacobian determinants). Additionally, the association between PRS and these morphometric markers was tested. We used non-parametric permutation tests (10.000 permutations) using Permutation Analysis of Linear Models (PALM) (Winkler et al., 2014, 2015). Permutation tests account for multiple comparisons and PALM specifically restricts the shuffling of observations that cannot be permuted freely according to exchangeability blocks, which was necessary with the family structure (twins and non-twin siblings) in the HCP data (Winkler et al., 2015). Age, gender and education level were added as covariates. The results were family-wise error rate (FWER) corrected at *p* < 0.05. See Fig. 1 for an overview of the analyses.

#### 2.5.2 Resting-state fMRI

The subject-specific spatial map represents each subject’s functional co-activation of the DMN estimated at group level, expressed as voxel-wise regression coefficients. Non-parametric permutation tests were used to assess group differences in the subject-specific spatial maps of the DMN between APOE *ε*4-carriers and non-carriers, as well as association with PRS (Winkler et al., 2015). Permutation tests, according to exchangeability blocks, were performed with PALM. Adjacency between vertices of the cortical surface data was taken into account, because nearby vertices are not independent. We added age, gender, education level and head motion (mean framewise displacement) as covariates to the model. PALM was run separately for the left and right hemisphere and subcortical data (10.000 permutations, with TFCE inference), after which we combined the results. The results were FWER-corrected at *p* < 0.05. See Fig. 1 for an overview of the analyses.

#### 2.5.3 Task fMRI

**Fig. 2:**
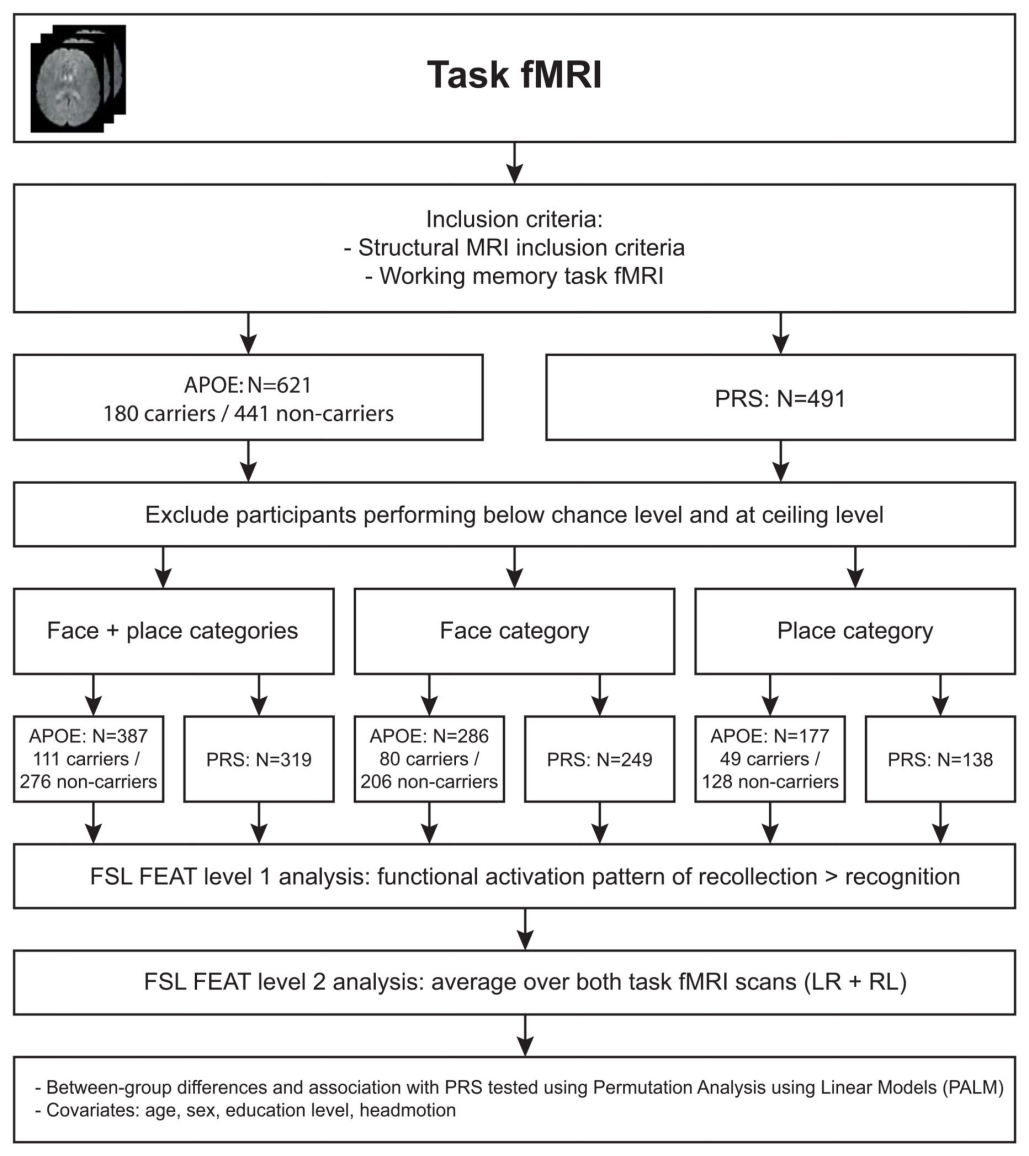
Schematic overview of the task fMRI analyses.

Recognition performance (old vs. new) was calculated using the d’ (d-prime) measure. Participants with a d’ score at or below zero were excluded, as this indicates a performance at or below chance level for old-new discrimination. The contrast of interest for the working memory task fMRI data was remember vs. know (i.e. recollection performance), which was analysed using FMRIB’s Expert Analysis Tool (FEAT) in FSL (v 6.0) (Woolrich et al., 2001). During the analysis phase, we additionally excluded participants performing at ceiling level for recollection performance, since this led to rank deficiency issues in FEAT^3^. The functional activation patterns of the encoding of the images leading to either recollection or recognition was determined using the results of the “Remember, Know, New” item recognition test and the available timing of these images during the 0-back task. As the categories of the item recognition tests belong to different semantic categories, we analysed the two categories of ‘face’ and ‘place’ images both together and separate. Higher level analyses for group differences or association with PRS were performed using PALM (10.000 permutations, with TFCE inference), as FMRIB’s Local Analysis of Mixed Effects (FLAME) cannot correct for the family structure in the HCP data. Age, gender, education level and head motion were included as covariates. The results were FWER-corrected at *p* < 0.05. An overview of the analyses can be seen in Figs. 1 and 2.

Filippini et al. suggested that increased DMN co-activation and increased task-related activation in APOE *ε*4-carriers might be a reflection of a compensation mechanism (Filippini et al., 2009). The HCP sample size permitted us to perform an additional test to compare DMN co-activation and task-related activation between APOE *ε*4-carriers and non-carriers. Under the hypothesis outlined in Filippini et al., APOE *ε*4-carriers need increased DMN co-activation or task-related activation for matched performance to non-carriers (Filippini et al., 2009). We therefore corrected the task fMRI analyses of the ‘face’ category for the score of the item recognition test to examine whether the compensation hypothesis holds^4^. In our analysis of the compensation hypothesis, we used all items from the item recognition task (both 0-back and 2-back tasks) across categories, and for faces and places separately to compute recollection scores. New items (foils) could compete with items from both the 2-back and 0-back tasks, which means that it is not possible to obtain a reliable estimate of recognition for each task independently.

These scores were computed as: Recollection = the proportion of “Remember” responses / 1 – the proportion of “Know” responses (Jacoby et al., 1997; Przeździk et al., 2019). Specifically, we computed the proportion of “Remember” and “Know” responses by dividing the number of correctly identified old items (i.e. the correct remember and know responses, respectively) by the total number of responses for that participant. Items to which participants did not respond were excluded from the analysis. Participants for whom one session was missing were also excluded. Statistical analyses of the neuroimaging data were similar to the resting-state fMRI and task fMRI analyses.

### 2.6 Quality checks and outcome-neutral validation tests

Extensive data quality checks were performed by the WU-Minn HCP Consortium with multiple levels of quality control throughout data acquisition (Marcus et al., 2013). The gray matter volumes and ICA components have been checked for errors (Marcus et al., 2013). Additionally, we excluded participants with anatomical anomalies and segmentation and surface errors in the data. Next to that, we performed an outcome neutral validation test for the recollection vs. recognition contrast of the task fMRI analysis. A whole-group analysis was performed, where we expected higher hippocampal activation during the encoding of images that are recollected, corresponding to our hypothesis that recollection relies on hippocampal-dependent processes.

## 3 Results

### 3.1 Structural MRI

#### 3.1.1 APOE carriership (preregistered)

To study whether APOE *ε*4-carriership influences morphometric features, we included 787 participants. The two groups consisted of 243 APOE *ε*4-carriers and 544 non-carriers. We assessed the T1-weighted scans using three types of morphometric analyses; gray matter density was determined with FSL-VBM, brain volumes were determined with FreeSurfer and local shape differences were assessed using the Jacobian determinants. No between-group differences were found for gray matter density (p>0.27, t<387.65), brain volumes (p>0.37, t<2.00) and local shape differences (p>0.26, t<380.76). Including covariates of age, gender and education level did not alter the results. Morphometric features were not different between carriers and non-carriers.

#### 3.1.2 Polygenic risk score (preregistered)

For the PRS analysis of the morphometric features, we included 625 participants of inferred European descent, with accurate detection of polygenic variants, and a quality-controlled T1-weighted structural scan. No associations of PRS, either with or without inclusion of APOE allelic variants, with gray matter density (p>0.43, t<296.33 and p>0.16, t<504.04, respectively), brain volumes (p>0.19, t<2.21 and p>0.15, t<2.44 respectively) and local shape differences (p>0.26, t<356.58 and p>0.84, t<209.31, respectively) were found using PALM. The inclusion of covariates of age, gender and education level did not alter the results. PRS was not found to be associated with morphometric features.

### 3.2 Resting-state fMRI

#### 3.2.1 APOE carriership (preregistered)

For the main analysis, we studied the hypothesis that APOE *ε*4-carriers show increased co-activation of the DMN. Of the 787 participants included in the structural MRI analysis, 586 participants also completed all four resting-state fMRI scans, which were reconstructed using the improved image reconstruction algorithm (r227). The groups included 167 APOE *ε*4-carriers and 419 non-carriers. No significant differences in DMN co-activation were found between APOE *ε*4-carriers and non-carriers (p>0.37, t<50.49). Including different combinations of covariates of age, gender, education level and head motion did not alter the results. Carriers were not found to have increased DMN co-activation compared to non-carriers.

#### 3.2.2 Polygenic risk score (preregistered)

To study the association of PRS with DMN co-activation, we were able to include 454 participants in the rfMRI analysis, who completed all four resting-state fMRI scans with the r227 image reconstruction algorithm. No association of PRS (with or without APOE variants) with DMN co-activation was found (p>0.26, t<58.51 and p>0.18, t<68.37 respectively). Results did not differ after including (different combinations of) covariates of age, gender, education level and head motion. DMN co-activation was not found to be influenced by PRS.

### 3.3 Task fMRI

#### 3.3.1 APOE carriership (preregistered and post hoc analyses)

To study functional activation patterns of recollection performance between carriers and non-carriers, we used the 0-back in-scanner working memory task, which served as the encoding phase for the post-scanner item recognition task. We included 621 participants, who completed both working memory scans, in addition to the inclusion criteria of the structural MRI analysis. 574 participants performed above-chance level (d’>0) in both the ‘face’ and ‘place’ categories of the 0-back task. There were no group differences in d’-score, before or after excluding participants that performed below-chance level. In addition, no group differences were detected in the excluded participants.

**Fig. 3:**
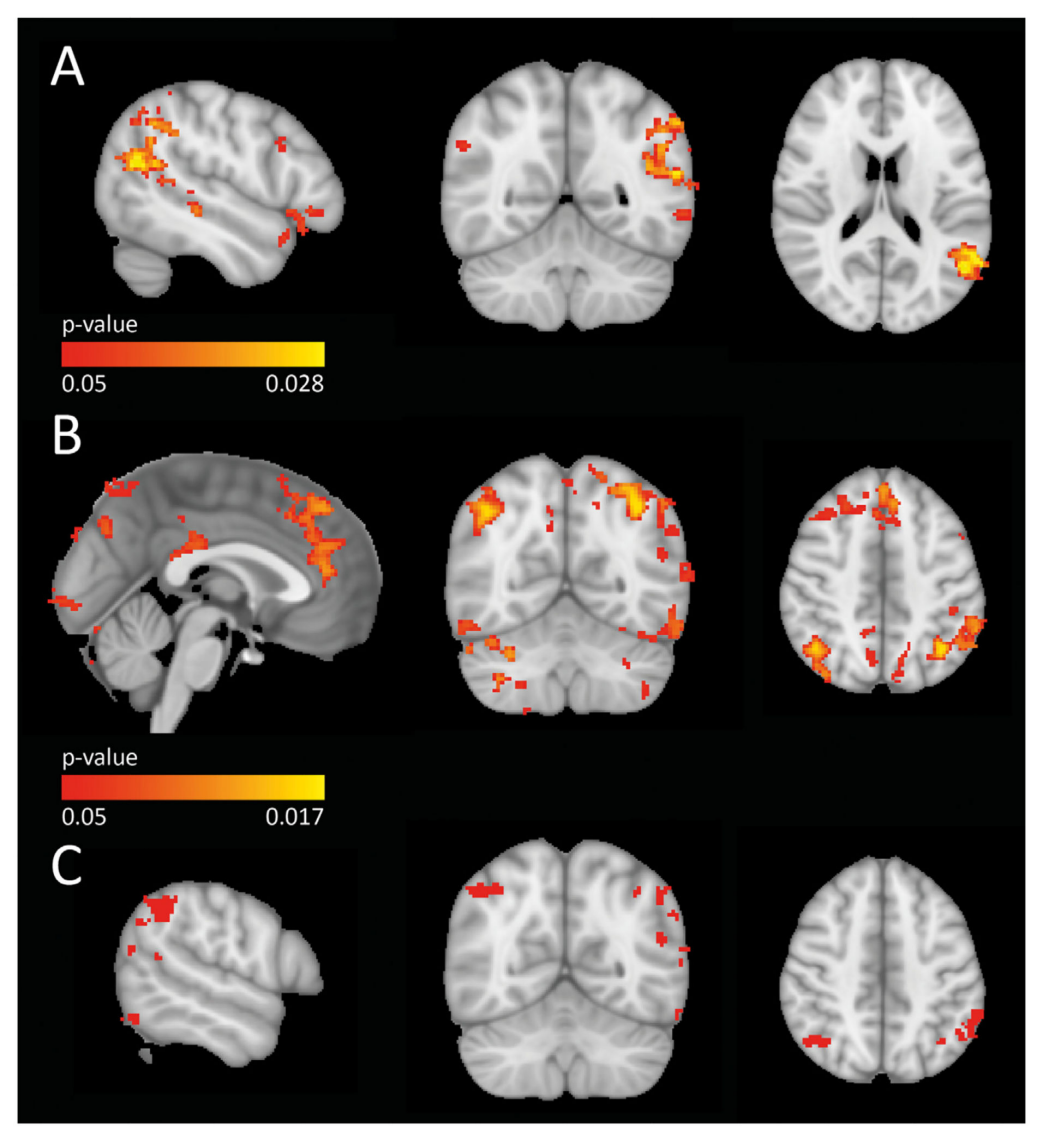
A: increased functional activation in APOE *ε*4-carriers over non-carriers found bilaterally in the lateral occipital cortex and angular gyrus, left middle temporal gyrus, left temporal pole and right middle frontal gyrus. B: increased functional activation associated with increased PRS detected in the superior frontal gyrus, left and right lateral occipital cortex, precuneous cortex and frontal pole. C: Overlap between increased functional activation of APOE *ε*4-carriership and higher PRS.

The functional activation pattern during the encoding of recollection vs. recognition was determined by linking the results of the item recognition task to the timing of the images during the N-back task in scanner. The other categories of the task were included as block regressors in the level 1 design. In the analysis of the combined categories of ‘face’ and ‘place’, 386 participants could be included, who did not perform at ceiling level in both scans. For the planned analysis of separate categories of ‘face’ and ‘place’, we included 286 and 177 participants, respectively. The higher-level group analyses of the combined categories ‘face’ and ‘place’ and the separate category ‘place’ did not show any between-group differences in functional activation during the encoding of subsequently recollected items (p>0.60, t<229.43 and p>0.34, t<305.05 respectively). However, in the higher-level analysis of the ‘face’ category we found a significant increased functional activation pattern in APOE *ε*4-carriers relative to non-carriers (p<0.05, t>756.11). This functional activation pattern differs from the initial findings of Filippini et al. (2009), with increased activation found bilaterally in the lateral occipital cortex and angular gyrus, left middle temporal gyrus, left temporal pole and right middle frontal gyrus (Fig. 3A, Table 1). Including different combinations of covariates of age, gender, education level and head motion did not alter the results. To study the compensation hypothesis, we performed a post hoc analysis, where we corrected the task fMRI analysis of the ‘face’ category for the effect of the score of the item recognition test. The score of the item recognition test was higher in APOE *ε*4-carriers (p=0.02, t=1.99), after adding the score as covariate to the task fMRI analysis, increased activation remained present (p<0.05, t>745.16). Functional activation patterns of recollection performance were found to be increased in *ε*4-carriers in the ‘face’ category, the activation patterns remained increased after correcting for the effect of recollection performance.

#### 3.3.2 Polygenic risk score (preregistered and post hoc analyses)

In addition, we studied the association of PRS with functional activation patterns of recollection performance. A subgroup of 491 participants of the structural MRI analysis also completed both working memory scans. We included 459 participants who performed above-chance level in the 0-back task in both the ‘face’ and ‘place’ category. We found a normal distribution of PRS in the group both before and after excluding participants (Fig. S1). In addition, PRS was not associated with D’-score (p>0.08).

For the analysis of the combined ‘face’ and ‘place’ categories, we included 319 participants who did not perform at ceiling level in both the LR or RL scan. For the separate analyses of the ‘face’ and ‘place’ category, 249 and 138 participants were included, respectively. We did not find any association of PRS with functional activation patterns during the encoding of subsequently recollected items in the combined categories ‘face’ and ‘place’ and the separate category ‘place’ (APOE variants included: p>0.26, t<339.57 and p>0.23, t<318.17 respectively, APOE variants not included: p>0.32, t<346.92 and p>0.25, t<208.74, respectively). In the analysis of the ‘face’ category we did detect an association of increased PRS (APOE variants included) with increased functional activation patterns during the encoding of subsequently recollected items (p<0.05, t>647.55 APOE variants not included: p>0.37, t<262.55). Increased activation is detected in the superior frontal gyrus, left and right lateral occipital cortex, precuneous cortex and frontal pole (Fig. 3B, Table 2). This functional activation pattern shows overlap with the findings from the APOE analysis (Fig. 3C). Including covariates of age, gender, education level, head motion and the first component from the multi-dimensional scaling analysis did not change the results. In our post hoc analysis on the compensation hypothesis, we corrected the task fMRI analysis of the ‘face’ category for the effect of recollection. A higher score of the item recognition test was associated with higher PRS (p=0.009, t=2.14). Increased activation remained present (p<0.05, t>616.15) after adding the score as covariate to the task fMRI analysis.

### 3.4 Quality checks

**Fig. 4:**
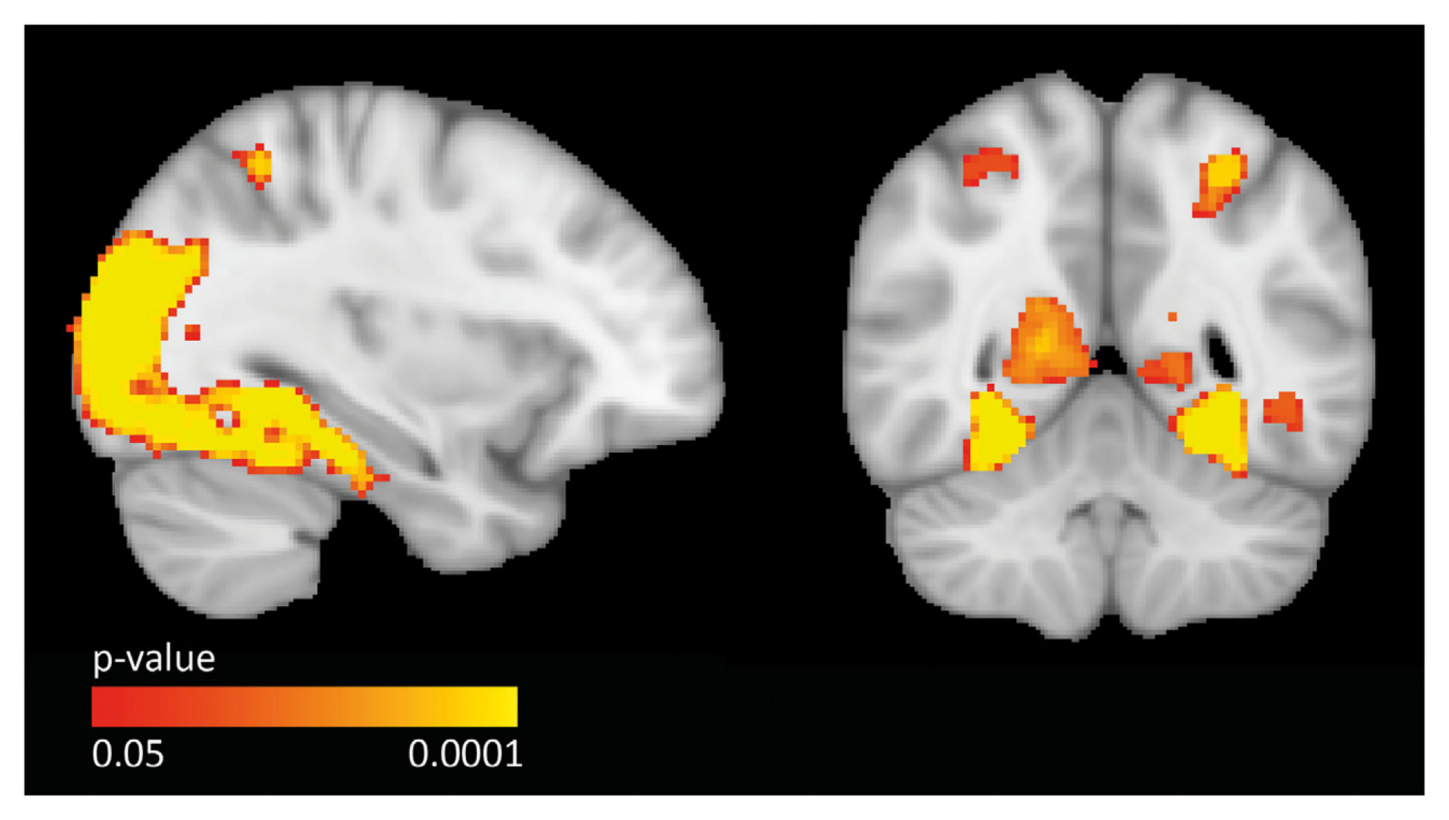
Whole-group functional activation pattern of recollection over recognition

Since we used an encoding task instead of the memory task used in the original study, we performed a whole-group analysis to ensure that encoding of images, which were afterwards recollected in the item recognition task, leads to higher hippocampal activation. We included the participants from the APOE analysis in both categories of ‘face’ and ‘place’ (n=386), and found that recollection vs. recognition led to significant activation in both the left and right hippocampus (p < 0.05, FWER corrected), as shown in Fig. 4.

## 4 Discussion

In this work, we aimed to replicate the early results of Filippini et al. (2009) in a substantially larger sample. In addition, we extended our replication study with a PRS-based analysis to understand whether polygenic contribution of common genetic variants to AD may influence brain structure and function in young adults. We used the WU-Minn HCP (S1200) dataset to analyse morphometric markers (brain volumes, gray matter density and local shape differences), DMN co-activation and working memory task-based activation. These features were assessed for differences between APOE *ε*4-carriers and non-carriers and for their association with PRS.

In contrast to Filippini et al. (2009), we did not find increased DMN co-activation and increased hippocampal task-based activation in APOE *ε*4-carriers relative to non-carriers. In addition, we did not find any significant group differences in brain volumes and other morphometric markers. We broadened the scope of our replication by calculating a PRS, both with and without the APOE variants. We did not find an association between PRS and morphometric markers, DMN co-activation and hippocampal task-based activation. Thus, our study on APOE *ε*4-carriership and polygenic risk for AD failed to replicate the early findings of Filippini et al. (2009). However, we did find increased task-based activation in APOE *ε*4-carriers and participants with higher PRS in other brain areas, which could indicate that genetic risk for AD modulates brain function in young adults. Nevertheless, task-based activation outcomes vary widely between studies (Dennis et al., 2010; Filippini et al., 2009; Matura et al., 2014; Mondadori et al., 2006), which impedes the use of these neuroimaging biomarkers for early risk assessment in clinical and research settings.

Very few studies have investigated the influence of genetic pre-disposition for AD on DMN co-activation in young adults. Apart from Filippini et al. (2009), Su et al. (2015) have also shown an increase in DMN functional connectivity compared to APOE *ε*3 and *ε*2-carriers, while others did not find any differences between *ε*4 and *ε*3-carriers (Dowell et al., 2016). In line with Dowell et al. (2016), we did not detect any group differences in DMN co-activation in young adults. The previous work has yielded mixed results in healthy young adults in the age range of 18-35 years old, using similar methodological protocols, although using small sample sizes (18-98 participants) from selective groups. Our study sample, representing the population at large, relevantly adds to these results. This outcome is not specific to APOE, but extends to the general trend of candidate gene studies in the field of behavioural genetics, which have usually failed to replicate the role of single genes in larger samples (Flint and Munafò, 2013).

In the task fMRI analysis, we studied recollection performance, by analysing the functional activation patterns during encoding of images that subsequently were recollected or recognized. We demonstrated that encoding of images leading to recollection over recognition was associated with activation in the MTL structures. While we did not replicate the findings of Filippini et al., with increased task activation in the hippocampal areas, we did find increased activation in APOE *ε*4-carriers relative to non-carriers during the 0-back face task in areas related to facial recognition, such as the lateral occipital cortex and the area surrounding superior temporal sulcus containing the fusiform face area (Freiwald et al., 2016). Even though APOE *ε*4-carriers had a higher recollection performance than non-carriers, when controlling for this effect, increased task activation was still detected. Although we found increased task activation in other areas, this could mean that APOE *ε*4-carriers need higher activation to recollect faces, indicating towards the compensation hypothesis proposed by Filippini et al. (2009) and Matura et al. (2014). Our findings add to the wide variation in outcome shown in earlier studies on task-based activation: APOE *ε*4-carriers were found to have both increased (Dennis et al., 2010; Filippini et al., 2009; Matura et al., 2014) and decreased (Mondadori et al., 2006) hippocampal task-related activation compared to non-carriers during both encoding and memory retrieval tasks.

In line with the results from Filippini et al. (2009), we found no differences in morphometric features between the APOE *ε*4-carriers and non-carriers. While Filippini et al. (2009) focused on assessing brain volumes using VBM, we assessed morphometric features in depth, studying brain volumes, gray matter density and local shape differences. Like earlier research on DMN co-activation and task activation, outcomes varied widely across studies; some found no differences between APOE carriers and non-carriers (Filippini et al., 2009; Khan et al., 2014; Matura et al., 2014; Su et al., 2015), while others detected reduced hippocampal volume (Alexopoulos et al., 2011; O’Dwyer et al., 2012) or reduced entorhinal thickness (Shaw et al., 2007). However, these results could be driven by the larger hippocampal volume and entorhinal thickness found in *ε*2-carriers, who are not included in our study (Alexopoulos et al., 2011; Shaw et al., 2007). Consequently, although we are currently unable to make any conclusive remarks, the results are supportive for none to limited genetic effects on morphometric features in young, healthy adults.

We extended our replication with a PRS analysis, which allows for data-driven investigation of the aggregated effects of many common risk variants as determined in a large European GWAS (Chasioti et al., 2019; Jansen et al., 2019). AD case-control studies show increased prediction accuracy when using PRS, identifying high-risk individuals beyond APOE *ε*4-carriership (Chouraki et al., 2016; Desikan et al., 2017; Escott-Price et al., 2015; Tan et al., 2017). We used the summary statistics of the large GWAS by Jansen et al. (2019), who included clinically diagnosed AD patients, as well as ‘AD-by-proxy’ cases, based on parental diagnoses of AD, who show a strong genetic correlation with AD (r_g_=0.81). They estimated the PRS with APOE explains 7.1% of the variance in clinical AD and PRS without APOE 3.9%. Despite this increased sensitivity, few studies have been performed on the association between polygenic risk and AD imaging phenotypes. In older adults, increased polygenic risk was associated with reduced hippocampal volume (Foo et al., 2021; Lupton et al., 2016) and entorhinal cortex volume (Desikan et al., 2017) as well as annual cortical and hippocampal thinning rates (Harrison et al., 2016; Sabuncu et al., 2012). First studies in healthy young adults reported an association between higher PRS and decreased hippocampal volume (Foley et al., 2017; Walhovd et al., 2020) and decreased left precuneus volume (Li et al., 2018). These results are in contrast with our findings, using one of the largest GWAS for AD to date. Unlike the previous studies in young adults, all using the AD case-control GWAS by Lambert et al. (2013), the GWAS summary statistics we used to build our PRS include ‘AD-by-proxy’ individuals. Despite the strong correlation between AD diagnosis and ‘AD-by-proxy’ cases based on parental diagnoses, our non-significant results may be a consequence of the ‘AD-by-proxy’ cases. By including these participants, who will not necessarily be diagnosed with AD in later life, we may have included irrelevant genetic factors in the analysis, leading to additional noise. Furthermore, since PRS-AD associations are more consistently found in samples with older adults, and the lifespan analysis by Walhovd et al. (2020) could not exclude an aging effect, we could speculate that risk for AD is driven by genetic factors whose effects become more pronounced at a later stage.

While the original findings could be a false positive, meaning that genetic risk for AD does not modulate brain function at a young age there are also several possible explanations for the failure to replicate the original findings of Filippini et al. (2009). Firstly, there are methodological differences between our study and Filippini et al. (2009) even though we adhered to their methods as much as possible. The specific deviations from the original study, such as advanced data preprocessing and choice of working memory task, were outlined in Section 2.1. Although not expected, these deviations, for example using an encoding task instead of a memory retrieval task, could have led to a failure to replicate the increased hippocampal activation in APOE *ε*4-carriers. Furthermore, our results could be related to lower SNR in the subcortical areas compared to Filippini et al. (2009), as the HCP favours higher spatial resolution in the trade-off between the two (Smith et al., 2013). Secondly, using a significantly larger study sample will likely result in increased heterogeneity in the sample, leading to a “regression to the mean” effect, where outcomes tend to be closer to the population mean (Held et al., 2020; Senn, 2011). Similar effects were established e.g. in the field of psychiatry, where, in patient vs. control prediction, accuracy of group delineation decreases with increasing sample size (Wolfers et al., 2015). If we are indeed dealing with regression to the mean and a true non-null finding, the previous findings of Filippini et al. (2009) are suggested to be specific to a subpopulation. Thirdly, in case Filippini et al. (2009) have truly discovered a non-null effect in their small sample of 36 participants, the observed effect size is likely inflated, also known as the “winner’s curse” (Button et al., 2013; Ioannidis, 2008). This impacts on replication studies, as the true effect is expected to be smaller. However, a true non-null effect is unlikely, as our large sample size provides power to detect much smaller effect sizes than the effect reported previously. Fourthly, as described above, there are limited studies on this subject, performed with mostly small samples, which show a wide variation in outcome. Expectedly, only few report no effects. We can only speculate as to whether we are dealing with publication bias, as negative findings in small studies are often rejected by journals, also known as the “file drawer problem” (Button et al., 2013).

In sum, we were not able to replicate the effect of APOE *ε*4-carriership on brain function in young adults. In addition, we broadened the scope of the original study by incorporating PRSs, thereby incorporating additional risk for AD beyond APOE *ε*4-carriership. Taking the methodological considerations of replication studies into account, the true effect of APOE *ε*4-carriership in the broader population is likely smaller than indicated in the original Filippini paper. Speculating on the effect being smaller or even not present, this would lead to several clinical implications.

The previous findings are generally cited to demonstrate that brain function is modulated in APOE *ε*4-carriers decades before clinical representation of AD (Hafkemeijer et al., 2012; Huang and Mucke, 2012; Jack et al., 2010; Liu et al., 2013). In studies with older adults, altered DMN co-activation is seen before other biomarkers, such as amyloid-B deposition measured in PET and CSF, appear (Jack et al., 2013; Sheline et al., 2010). Failure to replicate findings on early AD biomarkers such as the DMN impacts the understanding of the preclinical AD stage, currently thought to begin decades before clinical expression of the disease (Sperling et al., 2011). This is relevant as many drug trials including elderly subjects with AD have not been successful, and plead for new, longer trials with asymptomatic, at-risk individuals (Schneider, 2017). Thus, the understanding of this early preclinical stage is very important, since this group is targeted for primary prevention and prodromal therapeutic trials (Dubois et al., 2016; Sperling et al., 2011). However, solely focusing on (neuroimaging) biomarkers to unravel the mechanistic pathway of AD will probably not solve the AD conundrum. Moreover, having AD pathology does not equal having dementia and cognitive impairment (Hachinski, 2019). Therefore, further research should be more broadly focused on the interactions of genetic risk, biomarkers, aging and lifestyle factors over the life course, and sensitive functional neuroimaging as used here may help disentangling these complex interactions.

## Supplementary Material

Supplementary material associated with this article can be found, in the online version, at 10.1016/j.neuroimage.2021.118304.

Supplemental File

## Figures and Tables

**Table 1:** Peak clusters in APOE *ε*4-carriers relative to non-carriers.

Voxel coordinates peak cluster			Corresponding structure	Peak p-value, FWER-corrected	t-statistic
x	y	z			
70	32	45	33% Left Lateral Occipital Cortex, 29% Left Angular Gyrus	0.028	887.04
73	44	36	43% Left Middle Temporal Gyrus, 15% Left Superior Temporal Gyrus	0.034	848.90
67	73	27	37% Left Temporal Pole, 24% Left Frontal Orbital Cortex	0.041	808.85
23	70	55	55% Right Middle Frontal Gyrus	0.036	837.41
21	33	60	62% Right Lateral Occipital Cortex, 25% Right Angular Gyrus	0.039	818.94

**Table 2:** Peak clusters associated with increased PRS (APOE variants included).

Voxel coordinates peak cluster			Corresponding structure	Peak p-value, FWER corrected	t-statistic
x	y	z			
31	26	14	Cerebellum	0.017	859.97
48	78	57	33% Superior Frontal Gyrus, 29% Paracingulate Gyrus	0.025	777.50
63	33	59	46% Left Lateral Occipital Cortex, 17% Left Angular Gyrus	0.019	821.38
24	33	58	46% Right Lateral Occipital Cortex, 17% Right Angular Gyrus	0.020	813.14
46	25	55	16% Precuneous Cortex	0.033	715.48
30	87	53	90% Right Frontal Pole	0.032	729.37

## Footnotes

1 For the PRS analyses, we decided to include only participants from inferred European descent. We decided on this deviation before data observation as the genetic data of a European subset could be imputed using the European 1000 genomes sample. Further, the summary statistics by Jansen et al. are based on a European sample, leading to more reliable PRSs in a European subset of participants.

2 Commonly, phenotypic information is used to determine the optimal P-value for included SNPs in the PRS model. The imaging analyses in PALM are performed in a 4D format, which is not compatible with the PRS approach, for which a single phenotypic value per participant (1D vector) is needed. Therefore, instead of using phenotypic variance to determine the optimal P-value threshold of the PRS model, we used the optimal thresholds for AD prediction determined in the GWAS by Jansen et al.

3 This concerns a change in exclusion criteria. Participants performing at ceiling level led to rank deficiency issues in FEAT during the level 1 analysis and therefore had to be excluded before analysis could be performed.

4 Our results showed an increased task-related activation in the analysis of the separate “face” category. Therefore, as described in the preregistered report, we corrected for the recollection scores of the item recognition test. The calculation of these scores was not previously described in the preregistered report.
