# Supplementary material for: Functional co-activation of the default mode network in APOE ε4-carriers: A replication study
Source: Neuroimage. Author manuscript; Available in PMC 2026 Mar 28. (PMC7618942; doi:10.1016/j.neuroimage.2021.118304)
Supplement: Supplemental File [file EMS212888-supplement-Supplemental_File.pdf]

Opportunities for flexible electricity loads such as hydrogen production from curtailed generation: supplementary material

Tyler H. Ruggles[a]*, Jacqueline A. Dowling[b], Nathan S. Lewis[b], and Ken Caldeira[a,c]
[a]*Carnegie Institution for Science, Stanford, California, USA*
[b]*California Institute of Technology, Pasadena, California, USA*
[c]*Breakthrough Energy, Kirkland, Washington, USA*
*truggles@carnegiescience.edu

Contents

S.10 Techno-economic characteristics **S.2**

S.11 Modeled hydrogen production costs **S.5**

S.12 Cost sensitivity **S.5**

S.13 Demand response **S.9**
- S.13.1 Demand response limitations S.10
- S.13.2 Excluding demand response S.10

S.14 Aggregate wind and solar profiles **S.14**

S.15 Expansion of electricity system generation capacities **S.15**

S.16 Low-emissions drop-in gasoline replacement **S.16**
- S.16.1 System additions for electrofuel production S.16
- S.16.2 Electrofuel system model constraints S.18
- S.16.3 Cost of electrofuel production S.19
- S.16.4 Electrofuel system capacity factors S.19

S.17 Benchmark fuel consumption values **S.20**

S.18 Expanding system flexibility via power-to-gas-to-power **S.21**

S.18.1 Power-to-gas-to-power model and costs S.21

S.18.2 Power-to-gas-to-power results S.23

S.19 Additional discussion **S.27**

S.19.1 Existing electricity markets S.27

S.19.2 Modeled results scaled to the existing U.S. energy system . S.27

S.10. Techno-economic characteristics

The following details support the calculated cost and system component values included in our macro-scale electricity system model (Table S.4) coupled with a flexible load represented as an electrolysis facility. All costs were inflation-adjusted to 2019 USD values using Ref.[S.1] and U.S. Department of Labor Bureau of Labor Statistic consumer price index data. A capital recovery factor (Eq. 3) was used to determine the annualized capital cost for each capital expenditure using a discount rate of 7%.

The costs used for battery energy storage are taken from Ref.[36] The fixed capital investment cost in Table 2 was the central value from the provided range of 225 – 469 $/kWh$_e$ for a system with a power-to-energy ratio of 1:4. We divide by $\sqrt{0.9}$ to convert from nameplate energy capacity to usable energy capacity by assuming a 90% round trip efficiency with $\sqrt{0.9}$ losses charging and $\sqrt{0.9}$ losses discharging. The fixed O&M cost is calculated from the 0.25 $/kWh$_e$ annual O&M cost plus the annual cost of augmentation of 2.5% of initial fixed capital investment plus warranty of 0.8% of the fixed capital investment. The fixed O&M cost was also converted to a cost per usable energy capacity dividing by $\sqrt{0.9}$.

The electrolyzer is based on a plant design capacity of 50,000 kg$_{H_2}$/day.[40] The fully installed and configured electrolyzer plant cost is 118 M$_{2012}$ (138 M$_{2019}$), or 66,400 ($_{2019}$/h)/kg$_{H_2}$ produced. The electrolyzer plant costs can be split into the stack (47% of total costs) and balance of plant (BoP) costs (53% of total costs). The stack has an estimated 7 year lifetime while the BoP components have an estimated 40 year lifetime.[40] The fixed annual O&M costs are estimated at 3.55 M$_{2016}$ (3.80 M$_{2019}$) for the whole plant. The default NREL H2A PEM electrolyzer stack uses 49.23 kWh$_e$/kg$_{H_2}$ and is 67.7% efficient based on the LHV of hydrogen and 80.0% efficient based

	electrolyzer	compressor
fixed cap. investment $\left(\frac{\$}{kg_{H_2}/h}\right)$	63,000	920
fixed annual O&M $\left(\frac{\$}{kg_{H_2}/h}\right)$	1,800	180
assumed lifetime (yr)	7 stack, 40 balance of plant	15
fixed hourly cost $\left(\frac{\$}{h \cdot kg_{H_2}/h}\right)$	1.1	0.032
conversion efficiency	61% (LHV)	–
variable cost $\left(\frac{\$}{kg_{H_2}}\right)$	0	0

Table S.4: **Electrolysis facility costs and characteristics:** details of the electrolysis facility are included and are directly taken or derived from Ref.[40] All dollar values are in US$_{2019}$ with additional details provided in Section S.10. The "–" mark indicates the value is not applicable. Electrolyzer costs in dollars per kWh$_e$ are provided in Table 2.

on the higher heating value (HHV). In addition to power losses in the electrolyzer, power used for ancillary services in the electrolyzer plant totals $5.04\,\text{kWh}_e/\text{kg}_{H_2}$. The availability and cost of water for electrolysis was not included in the model.

A compressor was modeled to compress the hydrogen gas in preparation for storage or transportation. The NREL H2A default compressor has a design flow rate of $58,000\,\text{kg}_{H_2}$/day. The installed cost is 2.07 M$_{2016}$ (2.22 M$_{2019}$), or 917 ($_{2019}$/h)/kg$_{H_2}$. The fixed total annual O&M costs equate to 412,000 ($_{2016}$/h)/kg$_{H_2}$ (441,000 ($_{2019}$/h)/kg$_{H_2}$). The default compressor power requirement is $1,500\,\text{kW}_e$ to serve the design flow rate and the energy required to compress $1\,\text{kg}_{H_2}$ is $0.621\,\text{kWh}_e/\text{kg}_{H_2}$.

The electrolyzer, BoP, and compressor were combined together into the "electrolysis facility." The variable power costs of the electrolyzer, BoP, and compressor in our model were combined because they are all dominated by electrical power consumption. The value $\eta_{electro}$ (eq. S.22) is the efficiency to create and compressed hydrogen for these three components,

$$\eta_{electro} = \left(\frac{49.2\text{kWh}_e}{\text{kg}_{H_2}} + \frac{5.1\text{kWh}_e}{\text{kg}_{H_2}} + \frac{0.6\text{kWh}_e}{\text{kg}_{H_2}}\right)^{-1} \cdot \frac{\text{kWh}_{H_2}}{\text{kg}_{H_2}}, \tag{S.22}$$

where $\eta_{electro} = 60.7\%$ based on the LHV of hydrogen or $\eta_{electro} = 71.8\%$ based on the HHV.

System flexibility types	Temporal flexibility	Technology examples
**Dispatchable Generation:** Achieved via generators technically and economically capable of load-following. Generation flexibility can operate at daily, weekly, or monthly scales.[a]	within a day	large-scale hydroelectricity,[S.2,b]<br>natural gas combined cycle[S.3]<br>with and without CCS,[S.4]<br>ethanol combined cycle[S.5]
	within a week	natural gas combined cycle[S.3]<br>with and without CCS,[S.4]<br>ethanol combined cycle[S.5]
	within a month or more	natural gas combined cycle[S.3]<br>with and without CCS,[S.4]<br>ethanol combined cycle[S.5]
**Energy Storage:** These technologies enable the capture of energy produced at one time for use at a later time. Energy availability can be temporally shifted with storage to later hours, days, weeks, months, or further into the future.	within a day	Li-ion, redox flow, and lead-acid batteries,[16]<br>flywheels and capacitors,[16]<br>thermal energy storage,[S.6]<br>pumped hydro storage,[57,S.7,S.8]<br>compressed air energy storage[S.9]
	within a week	thermal energy storage,[S.6]<br>pumped hydro storage,[57,S.7,S.8]<br>compressed air energy storage[S.9]
	within a month or more	pumped hydro storage,[57,S.7,S.8]<br>compressed air energy storage,[S.9]<br>power-to-gas-to-power[16,24,56,57,c]
**Load Flexibility:** These technologies are able to shift their energy usage either earlier or later in time at daily, weekly, or monthly scales. Flexible loads may be coordinated within demand response programs that can also incorporate voluntary reductions, within constraints, of non-critical loads.	within a day	air conditioning,[S.10]<br>smart appliances,[S.11,17]<br>electric vehicle charging,[S.12,S.13,5]
	within a week	pre-heating and pre-cooling larger commercial systems, generating synthetic fuels for heavy-duty trucking, industry, heating, etc.
	within a month or more	generating synthetic fuels for heavy-duty trucking, industry, heating, etc.

Table S.5: **Example system flexibility options**: flexible options include dispatchable generation, energy storage, and load flexibility. Dispatch flexibility and temporal shifts in energy load and energy storage can occur at day, week, and month scales. Technology examples of each system flexibility type and temporal shift scale are provided in the table. A robust transmission system helps enable system flexibility and is not itself listed here.

[a]Coal and nuclear are excluded from the list of flexible generators because, in general, they rely on steam Rankine cycles and light-water reactors which have not historically been configured to rapidly ramp up and down;[S.14] further, environmental considerations weigh against coal, especially in the absence of carbon capture and storage, and economic considerations limit low-capacity operation of such facilities.
[b]Large-scale hydroelectricity facilities can have water and agriculture constraints that make it difficult to follow loads. In general, hydropower plants can load-follow on scales of multiple minutes to several hours.
[c]Power-to-gas-to-power (PGP) energy storage technologies with geologic hydrogen storage can store energy for use in later months, seasons, or even multiple years.[16]

S.11. Modeled hydrogen production costs

Table S.6 presents the contributions of electrolysis facility costs and power costs for hydrogen production. The cost of distributing hydrogen from a centralized production facility to end users includes considerations of the type of end use, the scale of hydrogen demand, and distribution methods. Ref.[55] quotes a cost of 10.50 $\$_{2005}/\text{kg}_{\text{H}_2}$ for hydrogen distribution from a centralized production facility to an urban market with 1% hydrogen penetration as a transportation fuel using gaseous truck delivery. Liquid truck and pipeline delivery costs were more expensive based on the 1% market penetration. The cost of delivery is less affected by the availability of otherwise unused and curtailed power (the cost of electricity for liquefying hydrogen for transportation can be substantial[55]), and therefore was excluded from our study.

cost ($\$/\text{kg}_{\text{H}_2}$)	Dispatch	Dispatch+ Renew+Storage	Renew+Storage
fixed cost electrolysis	1.16	1.16	1.17
power cost (marginal)	1.49	1.35	0.00
total cost (marginal)	2.65	2.51	1.17

Table S.6: **Hydrogen production costs**: Contributions to the total cost of hydrogen production per kg are shown as the flexible load fraction approaches zero. Costs are shown based on marginal costs.

S.12. Cost sensitivity

The sensitivity of the results was analyzed with respect to 25% and 50% cost reductions from the baseline fixed costs of generation and electrolysis technologies (Table 2; Fig. S.7). Although the cost of electricity varies depending on fixed costs, the general shape of each distribution is relatively robust. Each scenario contains a region at low flexible load fractions in which MC_{flex} asymptotes to a relatively low value. In addition, high flexible load fractions produce a region in which MC_{flex} asymptotes to a relatively high value, with a transition region in between these two limiting regions.

The electricity costs in the Dispatch and Dispatch+Renew+Storage scenarios were sensitive to reductions in the fixed cost of natural gas generation with CCS (Fig. S.7(a,b)). Of the three generation technologies included in the Dispatch+Renew+Storage scenario (dispatchable natural gas generation

with CCS, wind, and solar), electricity costs were most sensitive to reductions in the fixed cost of natural gas generation with CCS. Of the two generation technologies included in the Renew+Storage scenario (wind and solar), the electricity costs were most sensitive to reductions in the fixed cost of wind generation.

The cost of electricity was less sensitive to the fixed cost of the electrolysis facility compared to the fixed costs of the generation technologies. However, changes in the fixed cost of the electrolysis facility can change MC_{flex} (Fig. S.7(j,k,l)). The fixed cost of the electrolysis facility is a metric of the cost of load flexibility in the system. To be cost effective, a relatively expensive electrolysis facility must operate at a higher capacity factor than a relatively inexpensive facility. Therefore, lower fixed cost electrolysis facilities allow lower cost load flexibility that can operate cost-effectively at lower capacity factors.

Figure S.8 shows the sensitivity of hydrogen production costs to reductions in fixed costs. The features of each distribution were relatively similar over the modeled range of cost reductions. The total costs of hydrogen production were most sensitive to the dominant generation technology in each scenario.

The cost of the electrolysis facility can be sensitive to the fixed cost of the generation technologies. The least-cost generation mix is a function of the fixed costs, hence the total available generation profiles change with changes in fixed costs. This interplay resulted in different optimal operational capacity factors for the electrolysis facilities, affecting installed capacities. For example, the cost of the electrolysis facility in the Dispatch+Renew+Storage scenario was sensitive to reductions in the fixed cost of natural gas generation with CCS at high flexible load fractions (Fig. S.8(b)). Using baseline costs, dispatchable natural gas generation with CCS was not included in the least-cost system above a flexible load fraction of 0.79. However, as the fixed cost of natural gas generation with CCS was reduced, natural gas generation with CCS remained part of the generation mix at higher flexible load fractions, allowing the electrolysis facility to operate at an increased capacity factor, thus reducing its capacity and cost. The cost of the electrolysis facility in least-cost systems was most sensitive to changes in the fixed cost of electrolyzer capacity (Fig. S.8(j,k,l)).

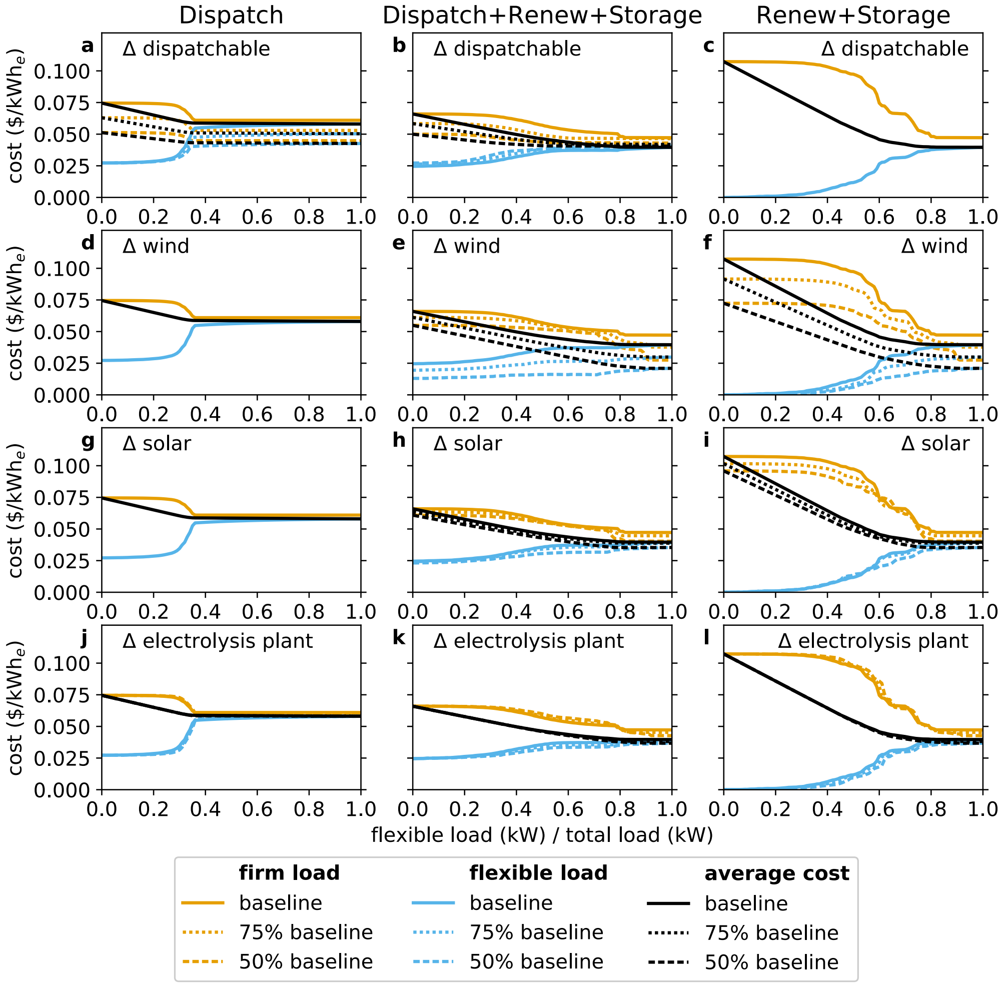

Figure S.7: **Sensitivity of electricity costs**: the sensitivity of electricity costs was assessed with respect to reductions in the fixed cost of natural gas with CCS, wind, and solar generation and electrolysis facility capacity. Electricity costs were attributed to the firm electricity and flexible electricity loads based on marginal costs (Section 2.3). The average cost of electricity is also shown. Panels are labeled indicating which technology cost was shifted.

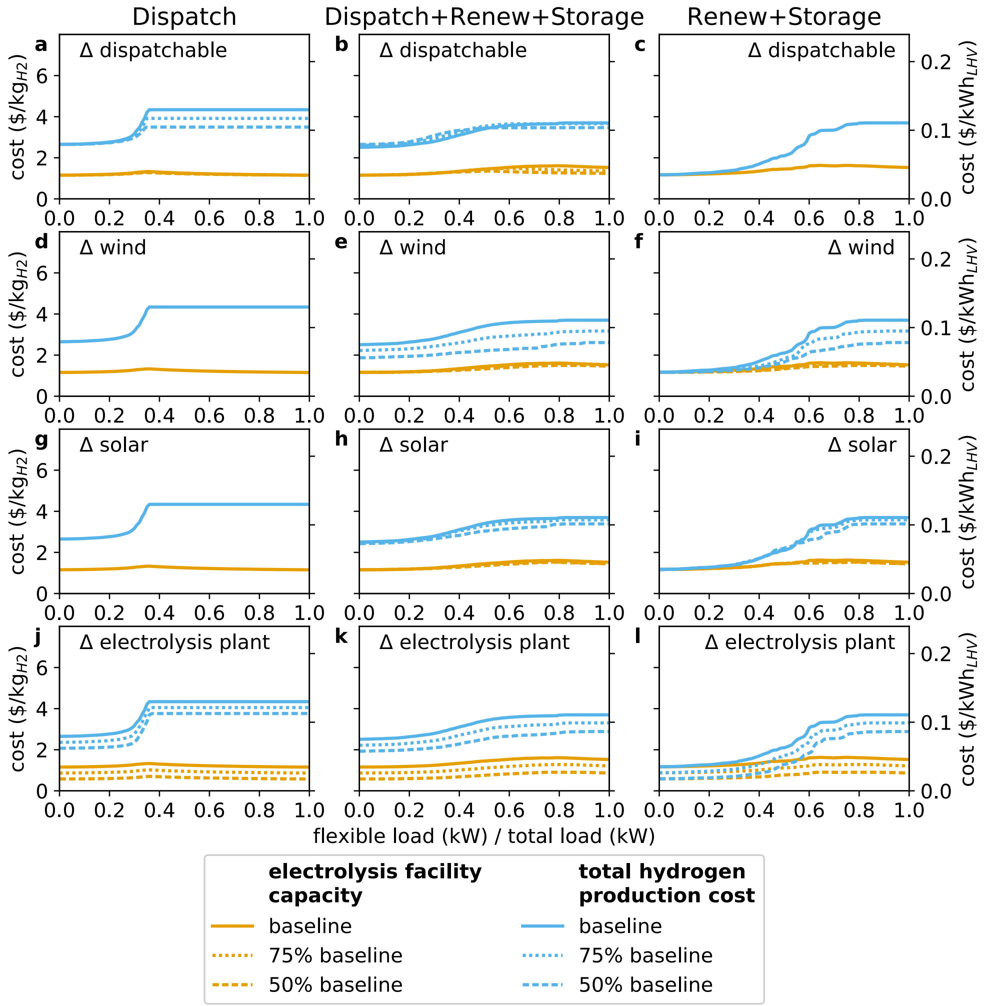

Figure S.8: **Sensitivity of hydrogen production costs**: The sensitivity of the hydrogen production costs were assessed with respect to reductions in the fixed cost of natural gas with CCS, wind, and solar generation and electrolysis facility capacity. The capacity-related costs of the electrolysis facility are shown in orange. Total hydrogen production costs (electrolysis facility capacity-related costs plus electricity costs) are shown using the marginal cost of electricity in blue (Section 2.3). Panel labels indicate which technology cost was shifted.

S.13. Demand response

A demand response mechanism was incorporated to represent a program in which customers would be paid to reduce their load during critical hours (no load shifting). In certain instances, the supplied firm load was less than the demanded firm load, and a cost proportional to the cost of demand response was incurred, 10 \$/kWh$_e$ (eqs. 13 and 16). The cost of demand response was based on the reported costs and energy savings for demand response programs in the U.S. in 2019.[50] The calculation used the ∼10% of reporting utilities that reported solely energy-based savings versus those that reported energy and capacity savings or solely reported capacity savings. This approach allowed a transparent calculation of dollars per kWh$_e$. The utilities that only reported energy savings saved ∼60 GWh$_e$ of energy, at a cost of \$400M, resulting in a cost of demand response of 7 \$/kWh$_e$, which we rounded to 10 \$/kWh$_e$.

Table S.7 shows the number of hours that experienced load reductions from demand response as well as the integrated total quantity of reduced load for the cases with zero flexible load. As the flexible load increased, the number of hours that experienced load reductions and the total quantity of reduced load decreased. Section S.13.2 describes results when 100% of the firm load was supplied at every hour (no demand response) (Figs. S.9, S.10, and S.11).

scenario	hours with demand response	hours with demand response (% per year)	maximum hourly reduction (% of peak firm load)	total load reduction (% of total firm electricity load)
Dispatch	27 h	0.31%	5.5%	0.012%
Renew+Storage	36 h	0.41%	38%	0.089%
Dispatch+ Renew+Storage	24 h	0.27%	6.0%	0.0061%

Table S.7: **Demand response**: the number of hours that experienced a reduction in the supplied firm load due to demand response, the maximum amount of load that was reduced, and the total annual quantity of load reductions as a percentage of annual firm electricity load are presented for the zero flexible load fraction simulations (total load is composed 100% of firm electricity load). The two scenarios with dispatchable generation, the Dispatch and Dispatch+Renew+Storage scenarios, used demand response less frequently and in smaller quantities.

S.13.1. Demand response limitations

Some demand response technologies are designed to reduce a load, while other demand response technologies shift the time of an electricity load. Shifting the firm load in time, instead of reducing it, would require that the load is eventually supplied. Thus, the quantity of unused and curtailed generation available for other flexible loads would decrease compared to what is modeled here. Table S.7 shows that the demand response mechanism was used during less than 0.5% of hours and reduced the total supplied firm load by less than 0.1% in all scenarios for the cases with zero flexible load. Shifting the less than 0.1% of firm load that was reduced would have a minimal impact on the total quantities of unused and curtailed generation used by the flexible load in this study (greater than 33% of generation was unused in all cases with zero flexible load, Section 3.1).

S.13.2. Excluding demand response

When 100% of firm load must be supplied every hour the system flexibility is reduced, and more generation capacity is installed (compare Figs. 2 and 3 with the versions that exclude demand response in Figs. S.9 and S.10). The cost of supplying electricity to the firm load (compare Fig. 2 with version that exclude demand response in Fig. S.9) as well as the resulting cost of producing electrolytic hydrogen (compare Fig. 5 with the version that exclude demand response in Fig. S.11) are consequently increased. Although the exact values change in these cases, the general conclusions of this study are robust and demonstrate that the demand response mechanism that increases the flexibility of the previously firm load reduces system costs, as expected.

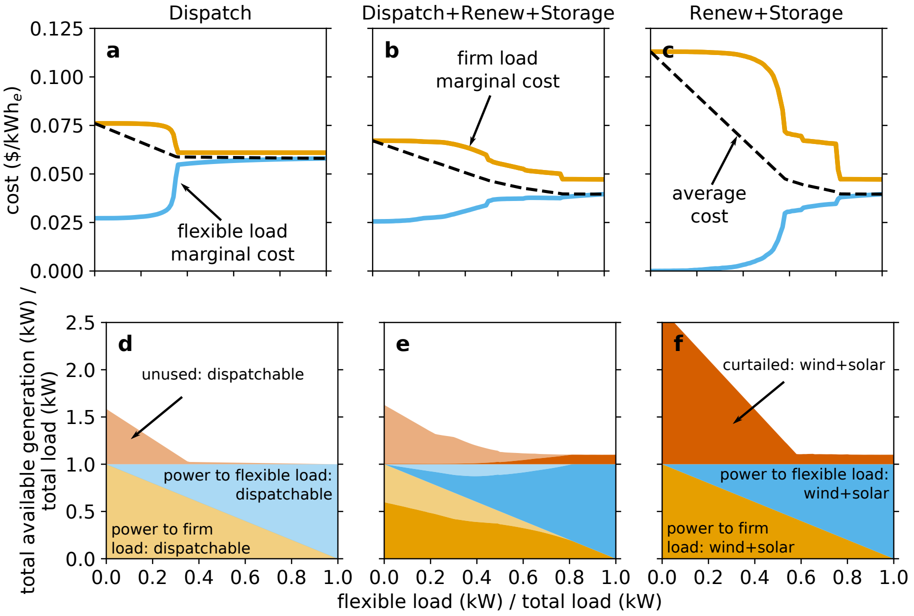

Figure S.9: **Electricity costs and generation end use by generation type - no demand response**: for an electricity system where 100% of firm electricity load is satisfied every hour the marginal cost of electricity that supplied the firm electricity and flexible electricity loads and the average cost of delivered power are shown across the full range of flexible load fractions in panels (a), (b), and (c). Total available generation is split into power used by the firm and flexible loads and unused and curtailed generation in panels (d), (e), and (f). The energy losses due to battery storage are not shown because they are negligible in the three scenarios. Compare against Fig. 2 where demand response is allowed for a cost of 10 $/kWh$_e$.

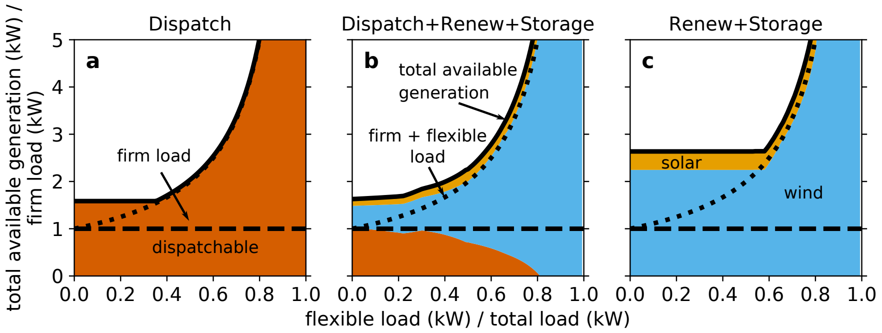

Figure S.10: **Electricity system generation capacities - no demand response**: for an electricity system where 100% of firm electricity load is satisfied every hour the total available generation capacity divided by the firm electricity load is shown across the flexible load fraction range for each generation type. This behavior shows the rate of generation expansion as a flexible load was added to a system that had a constant firm load. Compare against Fig. 3 where demand response is allowed for a cost of 10 \$/kWh$_e$.

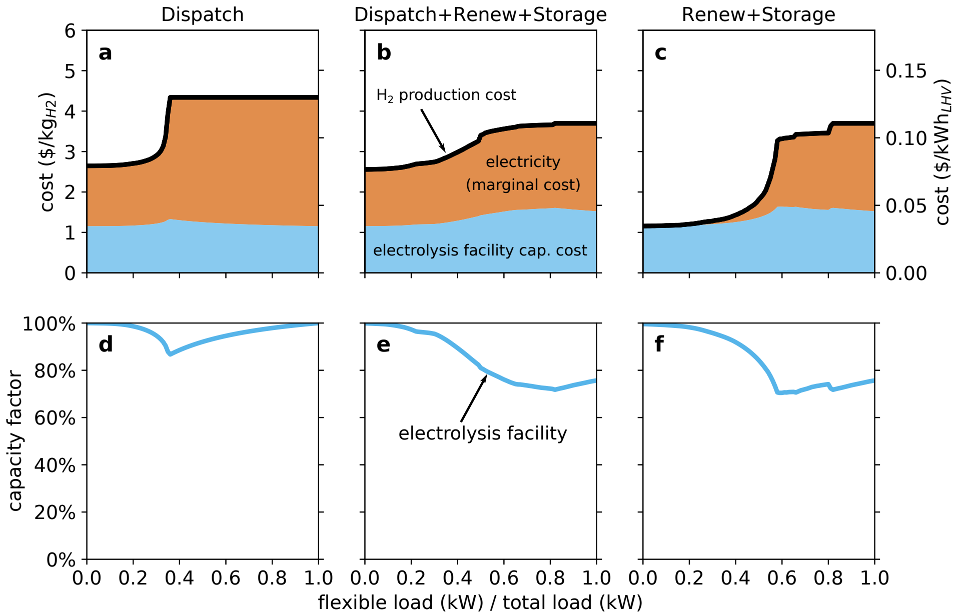

Figure S.11: **Hydrogen production costs and electrolysis facility capacity factor - no demand response**: for an electricity system where 100% of firm electricity load is satisfied every hour the cost of producing hydrogen per kg$_{H_2}$ and per kWh$_{LHV}$ is shown across the full range of flexible load fractions in panels (a), (b), and (c). The costs are split into the fixed cost of an electrolysis facility and the cost of electricity based on the marginal cost of electricity for a flexible load. For each scenario the electrolysis facility capacity factor is shown across the flexible load fraction range in panels (d), (e), and (f). The distributions show the near 100% capacity factor at low flexible load fractions, which began to decrease as the flexible load fraction increased. Compare against Fig. 5 where demand response is allowed for a cost of 10 \$/kWh$_e$.

S.14. Aggregate wind and solar profiles

The geographic regions with the highest annual capacity factors were used to produce time series that represent both wind and solar resources in this study. An alternative modeling choice would involve representing many resource subregions and attributing a resource profile and capacity to each subregion. This approach would allow the optimizer to account for synoptic scale correlations and anti-correlations between resource availability in different regions. In this method the optimizer could effectively tune the net wind and net solar profiles (the sum of all subregion profiles times their optimized capacities).

Table S.8 shows the nameplate installed capacities, average total available generation, mean capacity factors, curtailed generation, and storage capacity for the zero flexible load Renew+Storage scenario comparing these two resource representation methods. With the distributed method:

1. installed capacities of both wind and solar generation decrease;
2. average total available generation decreases for both wind and solar;
3. the mean capacity factors decrease for both wind and solar generation;
4. the average total curtailed generation is approximately halved; and
5. the least-cost storage capacity is approximately halved.

Although the distributed method produced a large reduction in curtailed generation, the curtailed generation is still zero variable cost electricity and would result in zero marginal cost electricity for the flexible load. Using the distributed method would lead to an accelerated rate of generation expansion as the flexible load increases because of less otherwise curtailed generation being available (Sections 3.1 and 3.2). Thus the $\sim$ zero marginal cost electricity shown in Figure 2(c) for the baseline Renew+Storage scenario would not extend to as high of a flexible load fraction. However, the general trends and conclusions from this study would still be robust.

One aspect of modeling the distributed method in this way is that the optimized selection of cells in which to build capacity will be sensitive to the modeled year of data. The optimizer can find the exact cell that perfectly complements the firm load profile at each hour. However, the choice of optimal locations will vary each year due to different weather fluctuations and firm load profiles. This situation contrasts to the aggregate method that selects cells for incorporation in the profile based on the annual mean capacity factors, which are likely to be more robust against annual variation.

	baseline aggregate profile	distributed MERRA-2 cells
nameplate installed capacity - wind	4.0	3.1
average total available generation - wind	1.7	1.3
mean capacity factors - wind	0.43	0.41
nameplate installed capacity - solar	1.6	1.5
average total available generation - solar	0.45	0.36
mean capacity factors - solar	0.28	0.24
average total curtailed generation	1.2	0.65
storage capacity	0.52	0.27

Table S.8: **Resource aggregation comparison**: two resource aggregation methods are compared, the baseline method aggregating 25% of CONUS cells into a single resource profile and a distributed method where each of the $\sim 2,500$ MERRA-2 cells is represented by its own resource profile and installed capacity. Nameplate installed capacities, average total available generation, mean capacity factors, curtailed generation, and storage capacity are shown for the zero flexible load Renew+Storage scenario. All units are represented in kW_e per kW_e of mean U.S. firm electricity load except the capacity factors, which are unitless.

Both of these two resource representation methods can reduce the resulting variability of the final resource profiles by aggregating together smaller regions (cells).[51,52] However, the variability cannot be eliminated. Barring zero cost energy storage or load flexibility, curtailed power will be present in least-cost electricity systems that are 100% powered by wind and solar energy.

S.15. Expansion of electricity system generation capacities

Table S.9 provides numeric values supporting Figure 3 and shows how the least-cost system generation capacities change for systems with different levels of flexible load penetration. Thus, Table S.9 effectively shows the rate of generation capacity expansion as greater quantities of flexible load are requested of the system. The table does not indicate the rate at which individual generation technology capacities change.

relative increase in total available generation	Dispatch		Dispatch+Renew+Storage		Renew+Storage	
	flexible load fraction	flexible-to-firm ratio	flexible load fraction	flexible-to-firm ratio	flexible load fraction	flexible-to-firm ratio
0.1%	0.21	0.27	0.01	0.01	0.24	0.31
1%	0.29	0.41	0.06	0.06	0.30	0.43
10%	0.38	0.61	0.27	0.37	0.50	1.00
25%	0.46	0.85	0.42	0.72	0.58	1.38

Table S.9: **Expansion of electricity system generation capacities**: Considering a constant firm electricity load, the percent change in total available generation was calculated with respect to the zero flexible load fraction simulation. Four illustrative points are listed for each scenario indicating at what flexible load fraction the generation capacity has expanded by 0.1%, 1%, 10%, and 25%. Also included is the flexible-to-firm load ratio.

S.16. Low-emissions drop-in gasoline replacement

The temporally flexible load was represented as a hydrogen production system that contained two distinct components: 1) a water electrolysis facility that produced hydrogen and 2) a compressor that compressed the hydrogen for storage.

We studied conversion of the hydrogen into a low-carbon emissions liquid hydrocarbon drop-in gasoline replacement fuel. To achieve this, the system model described in Fig. 1 was extended. A hydrogen storage facility was used to store the produced hydrogen and a chemical plant was added to convert the hydrogen plus other feedstocks into an "electrofuel."

S.16.1. System additions for electrofuel production

A salt cavern was used for hydrogen storage. The storage acted as a buffer between when hydrogen was produced (unused generation capacity was available) and when hydrogen was used as a feedstock by the chemical plant. The baseline characteristics and costs for the salt cavern were taken from the default values in the NREL H2A model.[37,39,S.15]

The H2A default salt cavern has a fixed capital investment (FCI) of 7.43 M$\$_{2016}$ (7.96 M$\$_{2019}$) with a usable storage quantity of $1,160,000\,\mathrm{kg}_{\mathrm{H}_2}$. The cost includes start up costs such as filling the cavern with a base quantity of hydrogen. This results in an FCI of $6.86\,\$_{2019}/\mathrm{kg}_{\mathrm{H}_2}$ storage. Annual O&M costs of $\$_{2016}$ 582,000 ($\$_{2019}$623,000), or $0.537\$_{2019}/\mathrm{kg}_{\mathrm{H}_2}$ storage, are included in the H2A model. The variable cost of storing more hydrogen is dominated by the power use of the compressor. Therefore, we attribute a variable cost

of $0\$_{2019}/kg_{H_2}$ storage. The cavern lifetime was assumed to be 30 years.[S.15] Cavern details are compiled in Table S.10.

To produce drop-in synthetic gasoline, a chemical plant was used to convert hydrogen into electrofuel. The chemical plant is based on common technologies used in the petrochemical industry to benefit from economies of scale and decades of knowledge. The specific chemical plant configuration and costs are complied in Ref.[S.16] The three main components of the plant are: 1) a reverse water-gas shift (RWGS) reformer, 2) a Fischer–Tropsch (FT) reactor, and 3) a hydrocracker. The two chemical feedstocks are hydrogen and CO_2. To allow greater flexibility of our model, CO_2 was treated as a feedstock that was purchased at a per unit cost of 50\$/ton, which is commensurate with estimates of flue gas carbon capture.

The RWGS reformer is used to produce CO by converting CO_2 and H_2 to CO and H_2O through endothermic hydrogenation[S.17] based on equation S.23:

$$CO_2 + H_2 \rightleftharpoons CO + H_2O. \tag{S.23}$$

Following the production of CO in the RWGS reformer, the FT reactor is used to polymerize H_2 and CO into n-alkane hydrocarbon chains over a catalyst. The products include gases (CH_4 through C_4H_{10}), liquids (C_5H_{12} through $C_{20}H_{42}$), and waxes with chain lengths longer than C_{20}.[S.18] The desired FT reaction is described by equation S.24:

$$(2n+1)\cdot H_2 + n\cdot CO \rightarrow C_nH_{2n+2} + n\cdot H_2O. \tag{S.24}$$

The longer wax hydrocarbon products must be split into shorter chains to produce a drop-in electrofuel. This transformation is performed in the hydrocracker. The hydrocracker design used in ref.[S.16] is based on the hydrocarbon yield distribution in ref.[S.19] After cracking the longer wax hydrocarbon chains, the final product distribution by mass fraction is 5.7% hydrocarbon gas (5.1% C_4H_{10}), 93.9% liquid hydrocarbons, 0.4% wax hydrocarbons and can be considered a drop-in substitute for conventional gasoline.[S.16]

The chemical plant described in ref.[S.16] used a model based on a flow sheet simulation developed in Aspen Plus®. The plant produces 56,300 kg_{EF} per hour (where kg_{EF} is kg of electrofuel), (a continuous rate of 690 MW_{LHV} of electrofuel) based on $30,000\,kg_{H_2}/h$ and $234,000\,kg_{CO_2}/h$ feedstocks. The plant has a chemical conversion efficiency of the feedstocks into electrofuel of 68.2% based on the LHV of the feedstock and electrofuel product. The variable costs per unit of electrofuel produced is calculated to include all

maintenance costs, taxes and incentives, utilities, and clean water with each contributing 55%, 30%, 13%, and 1% respectively for a total variable cost of 0.847 $\$_{2015}/\text{kg}_{EF}$ (0.915$\$_{2019}/\text{kg}_{EF}$). The plant lifetime is 30 years.[S.16]

Costs and technical characteristics of the RWGS reformer are based on ref.[S.20] with a TPC_{RWGS} = 32 M$\$_{2015}$ (35 M$\$_{2019}$), where TPC is total purchase cost . Costs and technical characteristics of the FT reactor are based on ref.[S.21] with a TPC_{FT} = 202 M$\$_{2015}$ (218 M$\$_{2019}$). Costs and technical characteristics of the hydrocracker are based on ref.[S.22] with a $\text{TPC}_{cracker}$ = 32 M$\$_{2015}$ (35 M$\$_{2019}$). Ref.[S.16] used a $\text{SF}_{chem\,plant}$ = 4.60 based on ref.[34] leading to a FCI for the chemical plant of 23,500 $\$_{2019}/(\text{kg}_{EF}/\text{h})$. Chemical plant details are compiled in Table S.10.

	storage cavern	chemical plant
technology type	salt cavern[S.15]	RWGS reformer,[S.20] FT reactor,[S.21] hydrocracker[S.22]
fixed capital investment (FCI)	6.86 $\$/\text{kg}_{H_2}$	23,500 $\$/(\text{kg}_{EF}/\text{h})$
fixed annual O&M	0.537 $\$/\text{kg}_{H_2}$	all attributed in variable cost
assumed lifetime (yrs)	30	30
annualized capital cost (ACC)	1.09 $\$/\text{kg}_{H_2}$	1,890 $\$/(\text{kg}_{EF}/\text{h})$
fixed hourly cost	0.000124 $(\$/\text{h})/\text{kg}_{H_2}$	0.216 $(\$/\text{h})/(\text{kg}_{EF}/\text{h})$
conversion efficiency (η)	–	68.2% (LHV)
variable cost	0 $\$/\text{kg}_{H_2}$	0.915 $\$/(\text{kg}_{EF}/\text{h})$
self-discharge rate	0.01% per year[S.23]	–

Table S.10: Details of the hydrogen storage cavern and chemical plant in the electrofuel system. All dollar values are in $\$_{2019}$. $\text{kg}_{EF}$is kg of electrofuel produced. "–" indicates the value is not applicable.

S.16.2. Electrofuel system model constraints

The original system is described in Section 2. When electrofuels are the desired end product, the system adds the capacity of the hydrogen storage cavern in kg_{H_2} and the chemical plant in kg_{EF}/h and their hourly operational characteristics to the objective function. Additionally, the exogenous

constraint on the quantity of energy supplied to the flexible load is changed to a constraint on the annual quantity of electrofuel produced.

S.16.3. Cost of electrofuel production

Figure S.12 shows the production costs of electrofuels. The lowest dollar per unit energy cost among all cases is found in the Renew+Storage scenario with near-zero flexible load present using the marginal cost of power, and still has a cost greater than 5 \$/GGE (dollars per gallon of gasoline equivalent).

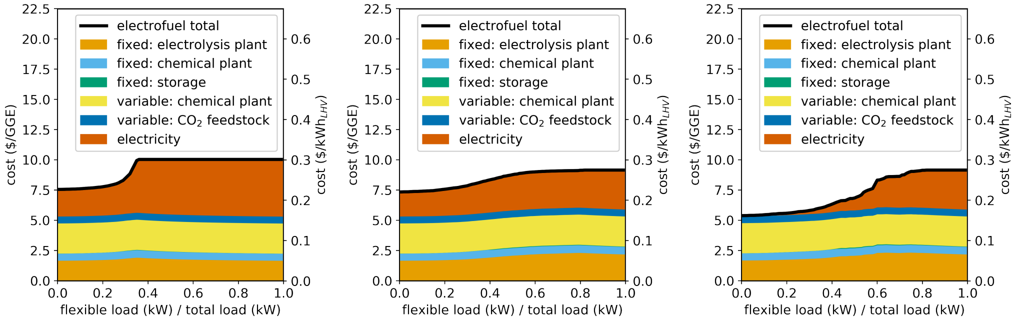

Figure S.12: **Electrofuel production costs**: the cost of producing an electrofuel per gallon of gasoline equivalent (GGE) and per kWh$_{LHV}$ is shown across the full range of flexible load fractions. The costs are split into the fixed and variable costs of the system components. The electricity cost for the electrolysis facility is shown based on the marginal cost of electricity for the flexible load. The Dispatch scenario is shown in panel (a), Dispatch+Renew+Storage in (b), and Renew+Storage scenario in (c).

S.16.4. Electrofuel system capacity factors

The electrolysis and electrofuel facilities capacity factors are shown in Fig. S.13. At low flexible load fractions, the distributions show near 100% capacity factors for the electrolysis plant and the chemical plant. In this range, no hydrogen storage capacity is built. Storage capacity is introduced at a flexible load fraction of ∼0.10 to 0.20 to decouple the operations of the electrolysis facility from the chemical plant, which allows the chemical plant to operate at a higher capacity factor compared to the electrolysis facility.

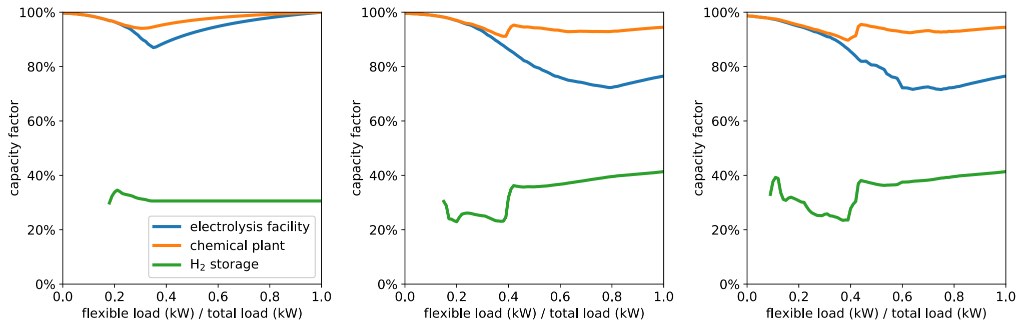

Figure S.13: **Electrolysis and electrofuel facilities capacity factors**: for each scenario the electrolysis facility, hydrogen storage cavern, and chemical plant capacity factors are shown across the flexible load fraction range. The Dispatch scenario is shown in panel (a), Dispatch+Renew+Storage in (b), and Renew+Storage scenario in (c).

S.17. Benchmark fuel consumption values

Three of the most difficult to decarbonize sectors are: long-distance road transportation, shipping, and aviation.[1] We provide an estimate for supplying the entire transportation sector with electrofuel.

In the U.S., aviation and shipping used an estimated 2,230 and 920 trillion Btu in 2017, while medium- and heavy-duty trucks used 1,390 and 4,900 trillion Btu. For comparison, in 2017 the total transportation sector consumed 26,800 trillion Btu (7,850 TWh).[S.24] These energy use values can be used for aviation and shipping but must be split into long-distance road transportation for medium- and heavy-duty trucks.

The U.S. Census Bureau estimates the miles driven by medium- and heavy-duty trucks based on their primary range of operation. Out of an estimated 145,000 million medium- and heavy-duty truck miles driven in the U.S. in 2002, $\sim$ 39% of those miles are attributed to trucks regularly taking trips greater than 100 miles.[S.25] Thus, 39% of energy use can be attributed to medium- and heavy-duty trucks for long-distance road transportation. The energy needs of the difficult-to-decarbonize portion of the transportation sector in the U.S. is the sum of aviation plus shipping plus 39% times (medium- and heavy-duty trucks), which equals 5,600 trillion Btu (1,640 TWh). This total, 5,600 trillion Btu, accounts for 21% of the total annual U.S. transportation energy use.

The modeled system requires approximately 4,000 TWh to produce 1,640

TWh of electrofuel because of the energy conversion efficiency of the electrolysis plant, 60.7%, and the chemical plant, 68.2%. This equates to a flexible load fraction of 0.50.

S.18. Expanding system flexibility via power-to-gas-to-power

Power-to-gas-to-power (PGP) technologies can add flexibility to electricity systems. In some cases PGP technologies can shift seasonal excess energy generation from variable wind and solar generation to later seasons when either production is lower and/or demand is higher.[56,16,24,57] Fuel cell and hydrogen storage technologies were introduced into the baseline model described in the Section 2 to assess how results varied with additional system flexibility.

S.18.1. Power-to-gas-to-power model and costs

Hydrogen storage was modeled as described in Section S.16.1 and in Table S.10. Fuel cell systems that recover and use a fraction of the heat generated during electricity production can exhibit efficiencies of 55%–80%.[S.26] We modeled a molten carbonate fuel cell with an efficiency of 70% and based the cost characteristics off of those presented in Ref,[S.26] 4,600 $\$_{2014}$/kW$_e$ (5,000 $\$_{2019}$/kW$_e$) with annual O&M costs of 40 $\$_{2014}$/kW$_e$ (43 $\$_{2019}$/kW$_e$). The same electrolyzer capacity that is used by the flexible load for hydrogen production in the baseline model is additionally used to produce hydrogen for the fuel cell. This synergy introduced a sharing of the fixed electrolysis facility capacity cost and capacity between the flexible load hydrogen demand and the hydrogen supplying the fuel cell. Fuel cell costs and characteristics are summarized in Table S.11.

The mathematical formulation of the model described in Section 2.2 was extended by adding a new storage technology component, s', that stores the produced hydrogen from the electrolysis facility with storage energy balance identical to eqns. 11 and 12. The rate of charging and discharging the hydrogen storage is limited by the electrolyzer and fuel cell capacities.

	fuel cell
technology type	molten carbonate fuel cell[S.26]
fixed capital investment (FCI)	5,000 \$/kW$_e$
fixed annual O&M	43 \$/kW$_e$
assumed lifetime (yrs)	20[S.27]
annualized capital cost (ACC)	510 \$/kW$_e$
fixed hourly cost	0.058 (\$/h)/kW$_e$
conversion efficiency (η)	70%
variable cost	0

Table S.11: Details of the modeled fuel cell used in the power-to-gas-to-power configuration of the model. All dollar values are in $\$_{2019}$.

$$0 \leq D_t^{\text{to s'}} \leq C^v \quad \forall t \tag{S.25}$$

$$0 \leq D_t^{\text{from s'}} \leq C^{v'} \quad \forall t \tag{S.26}$$

$$0 \leq S'_t \leq C^{s'} \quad \forall t \tag{S.27}$$

$$0 \leq D_t^{\text{from s'}} \leq S'_t(1-\delta^{s'}) \quad \forall t \tag{S.28}$$

where v denotes the electrolysis facility and v' denotes the fuel cell.

The same electrolysis facility capacity can act as the flexible load to generate hydrogen for an unspecified end use and produce hydrogen for use by the fuel cell, providing for a cost-sharing of the electrolysis facility capacity. To maintain a transparent cost allocation method, we attributed the cost of the electrolysis facility capacity proportional to the quantity of hydrogen consumed (eq. S.29).

$$\text{electricity system cost (PGP)} = \text{system cost} - \frac{D_{\text{flexible}}}{D_{\text{total}}} \sum_v c^v_{\text{fixed}} C^v \tag{S.29}$$

where D_{flexible} is the annual dispatch of hydrogen resulting from the flexible load and D_{total} is the total annual dispatch of hydrogen (flexible load plus fuel cell consumption). This cost allocation method affects the average and

marginal electricity costs shown in Figs. S.14(a,b,c) and S.16(a,b,c) and reduces to eq. 17 when zero fuel cell capacity is built. Thus, the average cost and marginal costs are calculated based on "electricity system cost (PGP)" (eq. S.29) instead of "electricity system cost" (eq. 17). This modifies eqns. 18, 19, and 21.

S.18.2. Power-to-gas-to-power results

The least-cost models only include PGP in the Renew+Storage cases, which represents the one scenario that does not involve a flexible, dispatchable generation technology. Fuel cell capacity is built when less flexibility is available in the total load; capacity is built in the Renew+Storage scenario for flexible load fractions from 0–0.55. The least-cost results are identical to those presented in the body of this paper when zero fuel cell capacity is built, such as for all flexible load fractions in the Dispatch and Dispatch+Renew+Storage scenarios.

The Dispatch and Dispatch+Renew+Storage cases produced identical results compared to their results when PGP was not included in the systems. This behavior results from the high cost of fuel cell capacity, (0.058 (\$/h)/kW$_e$ see Table S.11), relative to the lower dispatchable natural gas generation with CCS capacity cost (0.0308 (\$/h)/kW$_e$) and variable cost (0.027 \$/kWh$_e$ see Table 2).

Model results differ in the Renew+Storage cases, the only scenario without dispatchable generation, when PGP is allowed in the system:

1. The inclusion of PGP reduces the average cost of delivering electricity when zero flexible load is present in the system from 0.107\$/kWh$_e$ to 0.095\$/kWh$_e$, an 11% reduction (compare Fig. 2(c) against Fig. S.14(c)). The marginal cost of electricity for the flexible load remains at $\sim$ 0\$/kWh$_e$ when negligible flexible load is present.
2. Substantially less wind and solar generation capacity is overbuilt in the least-cost model when zero flexible load is present. The ratio of "total available generation"-to-"total load" when zero flexible load is present is reduced from 2.18 (a 118% overbuild) to 1.44 (a 44% overbuild) resulting in a 63% reduction in wind and solar generation overbuild by the inclusion of PGP (compare Fig. 2(f) against Fig. S.14(f)). "Total available generation" excludes fuel cell capacity.
3. The substantial reduction in curtailed wind and solar generation at low flexible load fractions leads to a more rapid increase in wind and

solar generation capacity as the flexible load fraction increases (compare Fig. 3(c) against Fig. S.15(c)).

4. The electrolysis facility capacity factor operates at $\sim 70\%$ when zero flexible load is present, representing a large difference compared to the $> 98\%$ capacity factor in the case without PGP (compare Fig. 5(f) against Fig. S.16(f)).

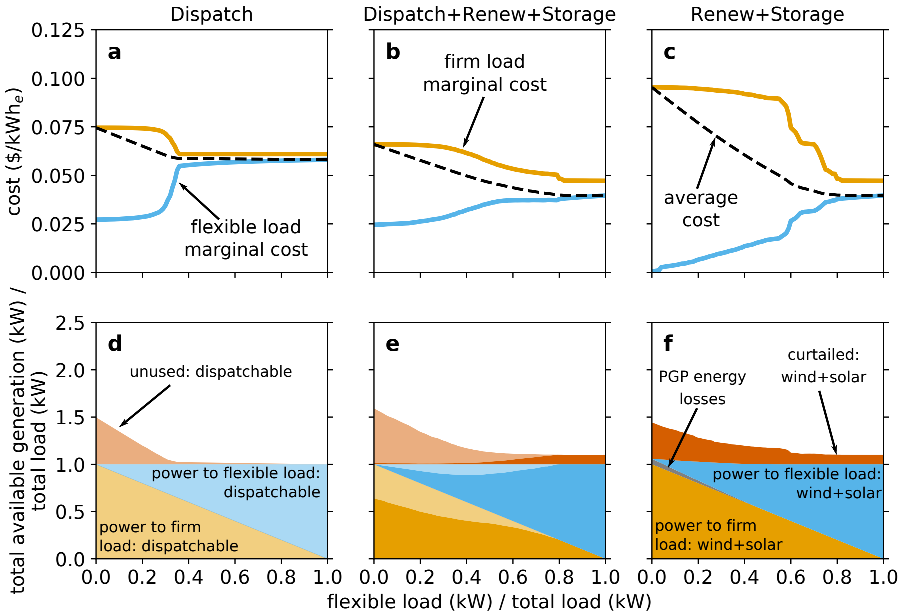

Figure S.14: **Electricity costs and generation end use by generation type - PGP results**: for an electricity systems that include power-to-gas-to-power (PGP) to increase system flexibility the marginal cost of electricity that supplied the firm electricity and flexible loads and the average cost of delivered power are shown across the full range of flexible load fractions in panels (a), (b), and (c). Total available generation is split into power used by the firm and flexible loads and unused and curtailed generation in panels (d), (e), and (f). The round-trip energy losses from PGP are visible in (f). The energy losses due to battery storage are not shown because they are negligible in the three scenarios. The fuel cell capacity is excluded from "total available generation". Compare against Fig. 2 where PGP is not included.

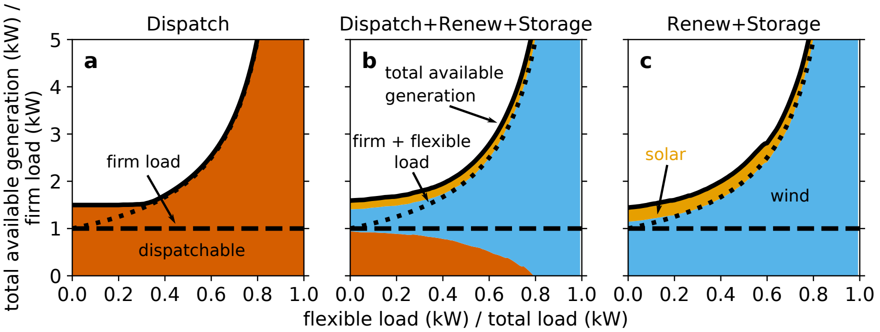

Figure S.15: **Electricity system generation capacities - PGP results**: for an electricity systems that include power-to-gas-to-power (PGP) to increase system flexibility the total available generation capacity divided by the firm electricity load is shown across the flexible load fraction range for each generation type. This behavior shows the rate of generation expansion as a flexible load was added to a system that had a constant firm load. The fuel cell capacity is excluded from "total available generation". Compare against Fig. 3 where PGP is not included.

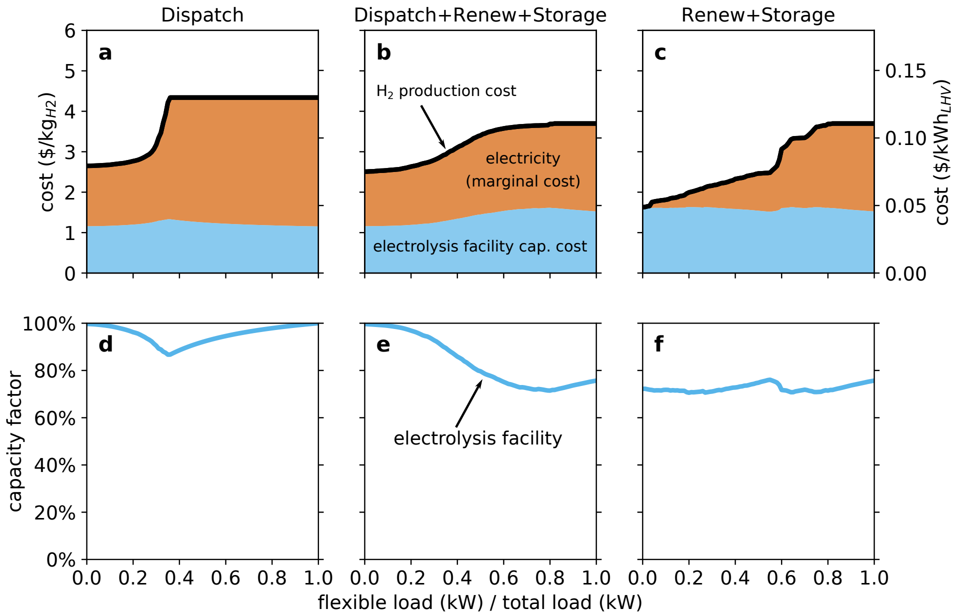

Figure S.16: **Hydrogen production costs and electrolysis facility capacity factor - PGP results**: for an electricity systems that include power-to-gas-to-power (PGP) to increase system flexibility the cost of producing hydrogen per kg_{H_2} and per kWh_{LHV} is shown across the full range of flexible load fractions in panels (a), (b), and (c). The costs are split into the fixed cost of an electrolysis facility and the cost of electricity based on the marginal cost of electricity. For each scenario the electrolysis facility capacity factor is shown across the flexible load fraction range in panels (d), (e), and (f). The distributions show the near 100% capacity factor at low flexible load fractions, which began to decrease as the flexible load fraction increased. Compare against Fig. 5 where PGP is not included.

S.19. Additional discussion

S.19.1. Existing electricity markets

Electricity consumers typically purchase electricity through retail electricity providers. Existing price structures for commercial and industrial consumers do not align exactly with the marginal cost method presented here (Section 2.3) nor do they perfectly align with average electricity costs.[26] The marginal cost method (MC_{firm} and MC_{flex}) distributes all electricity system costs based on time-of-use and the marginal cost during each hour. Most consumers do not have access to wholesale electricity markets with real-time marginal pricing.

Many retail electricity providers use tiered peak and non-peak hour pricing and demand charges to recover costs from commercial and industrial consumers.[26] As flexible loads are added to real electricity systems, otherwise unused and curtailed generation will have increasing uses, thus leading to a competitive market among different flexible loads, which would drive prices upward.

S.19.2. Modeled results scaled to the existing U.S. energy system

The results of our idealized model can be calibrated to the scale of the U.S. energy system for comparison. The addition of a substantial quantity of flexible loads will provide opportunities to use otherwise curtailed and unused generation, and lower average costs. Increasing load flexibility may have a positive feedback by reducing the amount of unused generation and enabling the cost-effective addition of even more variable renewable energy generation to electricity systems.

S.19.2.1. U.S. electricity system

The electricity load supplied by electricity systems in developed countries, including the U.S., is in general very inflexible. In 2018, electricity sales in the U.S. were approximately 4,000 TWh with Texas accounting for 11% of this total.[S.28] If our idealized models supplied 4,000 TWh of firm load annually, then annual unused generation would exceed 2,000 TWh in the Dispatch+Renew+Storage scenario with 4,000 TWh of curtailed generation in the Renew+Storage scenario (Fig. 2). Expanding generation capacity by 3% and 0%, respectively, in the least-cost models would allow Texas to double its electricity load if the new load were completely flexible (resulting in a flexible load fraction 0.10), while simultaneously reducing the average

cost of electricity by 6% and 10% for the Dispatch+Renew+Storage and Renew+Storage scenarios, respectively. Approximately 50 GW_e of electrolysis facility input capacity would be needed in both scenarios to enable this flexible load expansion, a capacity that far exceeds the 0.1 GW_e of installed PEM electrolyzer capacity worldwide.[S.29]

We also evaluated the amount of capacity expansion required to supply a given amount of flexible load. Adding 1,000 TWh of flexible load to an idealized U.S. electricity system that supplies 4,000 TWh of firm load annually could be achieved with minimal generation expansion (<0.1% in the Dispatch and Renew+Storage scenarios and an expansion of 6% in the Dispatch+Renew+Storage scenario). Thus, if the U.S. electricity system at this scale could be reasonably described by the modeled scenarios, ∼1,000 TWh of available generation could be used by a diverse mix of flexible loads, with minimal expansion of generation capacities (Figure 3).

The generation capacity of the U.S. electricity system is predicted to expand in the coming decades in part from economic growth, and in part from decarbonization and electrification of other energy sectors.[45] Studies show that in certain cases up to a doubling of generation capacity could be achieved by 2050 with respect to current infrastructure.[23] This doubling of generation capacity, largely through the addition of variable wind and solar resources, could roughly double the quantity of unused and curtailed generation available for flexible loads. If the future electricity system is highly regionalized and lacks the transmission necessary to smooth some of the variability in wind and solar availability, even more generation capacity may be needed, which in turn would lead to more unused and curtailed generation.

S.19.2.2. U.S. natural gas use

Hydrogen has been discussed as a potential replacement fuel for natural gas in certain circumstances.[S.30] A flexible load consuming power equal to that of Texas would produce 270 TWh of hydrogen annually, and constitute a small fraction (2.7%) of the 10,000 TWh of natural gas consumed by the U.S. in 2018.[S.31] However, many natural gas end uses can be electrified.[1] Given the present U.S. energy system, and assuming that residential use (1,500 TWh), commercial use (1,000 TWh), and use by the electricity sector (3,300 TWh) are all electrified, then 3,000 TWh of natural gas demand would remain in the industrial sector. 270 TWh of hydrogen could thus provide ∼10% of the more difficult to electrify industrial load. Regardless of the choice of denominator, the market for natural gas and potential natural gas replacements is large.

If flexible loads supply cost-competitive fuels to replace even a fraction of the existing natural gas market, future electricity systems could experience reductions in the average cost of electricity through the added load flexibility.

S.19.2.3. U.S. transportation fuels

Studies highlight the need for low-carbon fuels with high volumetric and gravimetric densities to address some of the most difficult to decarbonize sectors of the economy: long-distance road transportation, shipping, and aviation.[1] The crux of low-carbon, long-distance road transportation, shipping, and aviation is to maintain cargo space and payload capacity. Therefore, fuels with high volumetric and gravimetric densities are desired.[S.32] An extension of our model, described in Section S.16, produced such fuels. The annual fuel consumption of long-distance road transportation, shipping, and aviation is approximately 1,640 TWh in the U.S. (Section S.17). Approximately 4,000 TWh of electricity is need for electrolysis to generate 1,640 TWh of electrofuel because of energy conversion losses. An idealized system would supply the 4,000 TWh annual U.S. electricity load while also supplying 4,000 TWh to a flexible load producing hydrogen and a synthetic gasoline replacement in sequence. This system would have a flexible load fraction of 0.50 and a cost to produce electrofuels of $8.8 and $6.8 per gallon of gasoline equivalent, using the marginal cost of electricity for the Dispatch+Renew+Storage and Renew+Storage scenarios, respectively (Fig. S.12). The production cost includes a modest cost of 50 $/ton for CO_2 feedstock. Electrofuel production costs are substantial and will likely remain a barrier without the introduction of carbon pricing and/or technological breakthroughs.

References

[S.1] Coinnews Media Group LLC. Us inflation calculator. https://www.usinflationcalculator.com, 2020. visited on 2020-02-11.

[S.2] Hydropower vision: A new chapter for America's 1st renewable electricity source. Technical report, U.S. Department of Energy, 2016. https://doi.org/10.2172/1330494.

[S.3] TMI Staff & Contributors. Turbomachinery Magazine. Fast start combined cycle begins operation in California., 2012. https://www.turbomachinerymag.com/fast-start-combined-cycle-begins-operation-in-california/.

[S.4] Rubén M Montañés, Stefanía Ó GarÐarsdóttir, Fredrik Normann, Filip Johnsson, and Lars O Nord. Demonstrating load-change transient performance of a commercial-scale natural gas combined cycle power plant with post-combustion CO_2 capture. *International Journal of Greenhouse Gas Control*, 63:158–174, 2017.

[S.5] Ethanol power plant, minas gerais., 2010. https://www.power-technology.com/projects/ethanol-power-plant/.

[S.6] Dogan Erdemir and Ibrahim Dincer. Potential use of thermal energy storage for shifting cooling and heating load to off-peak load: A case study for residential building in canada. *Energy Storage*, 2(2):e125, 2020.

[S.7] Hossein Safaei and David W Keith. How much bulk energy storage is needed to decarbonize electricity? *Energy Environ. Sci.*, 8(12):3409–3417, 2015.

[S.8] J Paul Deane, BP Ó Gallachóir, and EJ McKeogh. Techno-economic review of existing and new pumped hydro energy storage plant. *Renewable and Sustainable Energy Reviews*, 14(4):1293–1302, 2010.

[S.9] Stephen A Rackley. *Carbon capture and storage*. Butterworth-Heinemann, 2017.

[S.10] WJN Turner, IS Walker, and J Roux. Peak load reductions: Electric load shifting with mechanical pre-cooling of residential buildings with low thermal mass. *Energy*, 82:1057–1067, 2015.

[S.11] Alain Moreau. Control strategy for domestic water heaters during peak periods and its impact on the demand for electricity. *Energy Procedia*, 12:1074–1082, 2011.

[S.12] Donna Hostick, David B Belzer, Stanton W Hadley, Tony Markel, Chris Marnay, and Michael Kintner-Meyer. Renewable electricity futures study. volume 3. End-Use electricity demand. Technical report, National Renewable Energy Lab. (NREL), 2012.

[S.13] Till Gnann, Anna-Lena Klingler, and Matthias Kühnbach. The load shift potential of plug-in electric vehicles with different amounts of charging infrastructure. *Journal of Power Sources*, 390:20–29, 2018.

[S.14] Arina Anisie and Francisco Boshell. Flexibility in conventional power plants: Innovation landscape brief. Technical report, International Renewable Energy Agency (IRENA), 2019. https://www.irena.org/-/media/Files/IRENA/Agency/Publication/2019/Sep/IRENA_Flexibility_in_CPPs_2019.pdf.

[S.15] Amgad Elgowainy, Krishna Reddi, Marianne Mintz, and Daryl Brown. H2A delivery scenario analysis model version 3.0*(HDSAM 3.0) user's manual, 2015.

[S.16] Daniel H. König, Marcel Freiberg, Ralph-Uwe Dietrich, and Antje Wörner. Techno-economic study of the storage of fluctuating renewable energy in liquid hydrocarbons. *Fuel*, 159:289 – 297, 2015.

[S.17] Rajabhau Bajirao Unde. *Kinetics and Reaction Engineering Aspects of Syngas Production by the Heterogeneously Catalysed Reverse Water Gas Shift Reaction*. PhD thesis, Bayreuth, 2012.

[S.18] Arno de Klerk. *Fischer–Tropsch Refining*. Wiley, 2011.

[S.19] Yanyong Liu, Kazuhisa Murata, Kiyomi Okabe, Megumu Inaba, Isao Takahara, and Toshiaki Hanaoka et al. Selective hydrocracking of Fischer–Tropsch waxes to high-quality diesel fuel over Pt-promoted polyoxocation-pillared montmorillonites. *Topics in Catalysis*, 52(6):597–608, 2009.

[S.20] Richard C Baliban, Josephine A Elia, and Christodoulos A Floudas. Toward novel hybrid biomass, coal, and natural gas processes for satisfying current transportation fuel demands, 1: Process alternatives, gasification modeling, process simulation, and economic analysis. *Industrial & Engineering Chemistry Research*, 49(16):7343–7370, 2010.

[S.21] Oscar P R van Vliet, André P C Faaij, and Wim C Turkenburg. Fischer–Tropsch diesel production in a well-to-wheel perspective: A carbon, energy flow and cost analysis. *Energy Convers. Manage.*, 50(4):855–876, 2009.

[S.22] W L Becker, R J Braun, M Penev, and M Melaina. Production of Fischer–Tropsch liquid fuels from high temperature solid oxide co-electrolysis units. *Energy*, 47(1):99–115, 2012.

[S.23] Fritz Crotogino, Sabine Donadei, U Bünger, and Hubert Landinger. Large-scale hydrogen underground storage for securing future energy supplies. In *18th World hydrogen energy conference*, volume 78, pages 37–45, 2010.

[S.24] Stacy C. Davis and Robert G. Boundy. Transportation energy data book: Edition 38.1. https://tedb.ornl.gov/wp-content/uploads/2020/02/TEDB_Ed_38_04302020.pdf, 2020. Oak Ridge National Laboratory.

[S.25] Census Bureau U.S. Department of Commerce. Vehicle inventory and use survey: United states: 2002. www.census.gov/prod/ec02/ec02tv-us.pdf, 2004.

[S.26] Ken Darrow, Rick Tidball, James Wang, and Anne Hampson. Catalog of CHP technologies. Technical report, US Environmental Protection Agency, 2015.

[S.27] D Steward, M Penev, G Saur, W Becker, and J Zuboy. Fuel cell power model version 2: Startup guide, system designs, and case studies. modeling electricity, heat, and hydrogen generation from fuel Cell-Based distributed energy systems. Technical report, National Renewable Energy Lab. (NREL), 2013.

[S.28] U.S. Energy Information Administration. State electricity profiles. https://www.eia.gov/electricity/state/, 2018. Release date: December 31, 2019.

[S.29] International Energy Agency. Hydrogen projects database. https://www.iea.org/reports/hydrogen-projects-database, 2020. visited on 2020-12-07.

[S.30] Joshua Eichman, Aaron Townsend, and Marc Melaina. Economic assessment of hydrogen technologies participating in California electricity markets. Technical report, National Renewable Energy Lab. (NREL), 2016.

[S.31] U.S. Energy Information Administration. Annual energy review. https://www.eia.gov/totalenergy/data/monthly/pdf/sec4_5.pdf, 2020. Section 6.5: Natural gas consumption by sector.

[S.32] Lewis M Fulton, Lee R Lynd, Alexander Körner, Nathanael Greene, and Luke R Tonachel. The need for biofuels as part of a low carbon energy future. *Biofuels Bioprod. Biorefin.*, 9(5):476–483, 2015.
